# Ergosterols from the Culture Broth of Marine *Streptomyces anandii* H41-59

**DOI:** 10.3390/md14050084

**Published:** 2016-05-04

**Authors:** Yang-Mei Zhang, Hong-Yu Li, Chen Hu, Hui-Fan Sheng, Ying Zhang, Bi-Run Lin, Guang-Xiong Zhou

**Affiliations:** 1 College of Pharmacy, Jinan University, Guangzhou 510632, China; yangmzhang@sina.com (Y.-M.Z.); hongyu88926@126.com (H.-Y.L.); kitten_hc@163.com (C.H.); carolynzy@163.com (Y.Z.); 2 Institute of Plant Protection, Guangdong Academy of Agricultural Sciences, Guangzhou 510632, China; shenhf@gdppri.com (H.-F.S.); linbr@126.com (B.-R.L.)

**Keywords:** *Streptomyces anandii*, ergosterols, strain identification, ananstreps A–C, cytotoxicity

## Abstract

An actinomycete strain, H41-59, isolated from sea sediment in a mangrove district, was identified as *Streptomyces anandii* on the basis of 16S rDNA gene sequence analysis as well as the investigation of its morphological, physiological and biochemical characteristics. Three new ergosterols, ananstreps A–C (**1**–**3**), along with ten known ones (**4**–**13**), were isolated from the culture broth of this strain. The gross structures of these new compounds were elucidated on the basis of extensive analysis of spectroscopic data, including HR-ESI-MS, and NMR. The cytotoxicities of these isolates against human breast adenocarcinoma cell line MCF-7, human glioblastoma cell line SF-268, and human lung cancer cell line NCI-H460 and their antibacterial activities in inhibiting the growth of *Candida albicans* and some other pathogenic microorganisms were tested. Compounds **3**–**8**, **10** and **11** displayed cytotoxicity with IC_50_ values in a range from 13.0 to 27.8 μg/mL. However, all the tested compounds showed no activity on *C. albicans* and other bacteria at the test concentration of 1 mg/mL with the paper disc diffusion method.

## 1. Introduction

In recent years, many researchers have paid attention to marine-derived microorganisms because of the increasing rate of rediscovery of known compounds on terrestrial land [1]. Marine microbial secondary metabolites are a prolific source of novel natural products, including sterols and other related metabolites with potent cytotoxic or antimicrobial activities [2,3]. Ergosterols are a class of components possessing many hydroxyl groups, a tetracyclic skeleton and a short alkyl chain. They are a sort of critical component in membranes, involved in many biological functions and play an important role in regulating fluidity of membrane, controlling cellular cycle, and organizing membranes for signal transduction and protein trafficking [4,5]. To the best of our knowledge, ergosterols were usually found in fungi, sponges, and corals, and it is rarely to discover new ergosterols from *Streptomyces* species [6,7]. *Streptomyces* are a genus of Gram-positive, filamentous bacteria usually dwelled in soil. They are one of the most diverse in species and show the ability to produce clinical useful compounds with different structures, such as streptomycins, actinomycins, adriamycin, vancomycin, and tacrolimus [8,9,10].

On our present study, a strain of actinomycete H41-59, isolated from sea sediment at a mangrove district of South China Sea, was identified as *Streptomyces anandii*. Application of multiple chromatographic procedures and modern spectral methods led to the isolation and identification of thirteen sterols including three new sterols (**1**–**3**). Their cytotoxicities against three cancer cell lines and their antibiotic activities inhibiting the growth of *C. albicans* and some pathogenic bacteria were evaluated. Herein, we describe the structure determination of three new compounds, the isolation and bioactivity assay of these isolated compounds from the ethanol extract of fermented broth of strain H41-59, and try to hypothesize the biosynthetic pathway of these sterols.

## 2. Results and Discussion

### 2.1. Characterization of the Compounds

**Figure 1:**
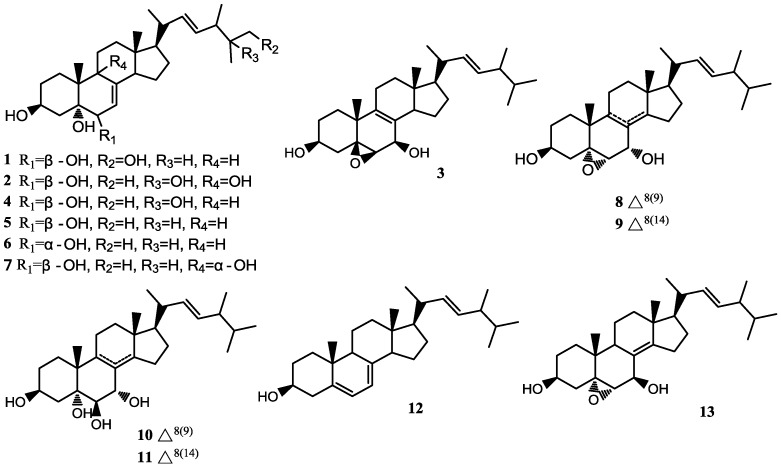
Structures of compounds **1**–**13**.

An ethyl acetate (EtOAc) partition from the 95% ethanol (EtOH) extract of mycelium of *Streptomyces anandii* strain H41-59 was subjected to repeated silica gel column chromatography and then purified by semi-preparative reverse phase HPLC separation to yield thirteen compounds (**1**–**13**, Figure 1). On the basis of the NMR analysis and the comparison with reported data, three of them were identified as new sterols (**1**–**3**).

Compound **1** was obtained as colorless needle crystal and gave a molecular formula of C_28_H_46_O_4_, deduced by the HR-ESI-MS [M + Na]^+^ ion at *m/z* 469.3293 (C_28_H_46_NaO_4_, calcd. 469.3288). There are two methyl singlets at *δ* 0.54 (Me-18) and 0.91 (Me-19), three doublets at *δ* 0.99 (Me-21, *J* = 6.6 Hz), 0.74 (Me-26, *J* = 6.9 Hz) and 0.92 (Me-28, *J* = 6.8 Hz), three olefinic protons at *δ* 5.08 (H-7, m), 5.19 (H-22, dd, *J* = 15.4, 7.9 Hz) and 5.21 (H-23, dd, *J* = 15.4, 7.1 Hz) in the ^1^H NMR spectrum. One oxymethylene at *δ* 64.4 (C-27), two oxymethines at *δ* 66.0 (C-3) and 72.1 (C-6), and an oxygenated quaternary carbon at *δ* 74.5 (C-5) existed in the ^13^C NMR spectrum, which were further confirmed by DEPT and HSQC spectra. All the spectroscopic data indicated that **1** was a tetrahydroxylated sterol, similar to those of the previously isolated compounds **4** and **5** except for the signals of the side chain.

**Figure 2:**
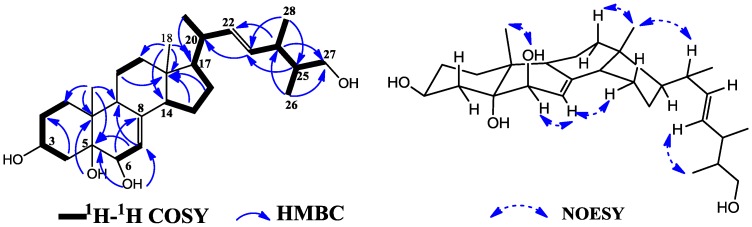
The key HMBC, H^1^-H^1^ COSY (**left**) and NOESY (**right**) correlations of **1**.

Compared with those of known compound **5** [11,12], there were visible changes at *δ* 36.9 (C-24), 40.5 (C-25), 13.1 (C-26) and 18.3 (C-28), and an additional oxymethylene at *δ* 64.4 (C-27). Position of hydroxyl group at C-27 was deduced by 2D NMR experiments (Figure 2). The methyl doublet at *δ* 0.74 (Me-26) displayed two HMBC correlations to C-24 (*δ* 36.9, *^3^J*) and C-27 (*δ* 64.4, *^3^J*), a methine multiplet at *δ* 2.15 (H-24) showed a HMBC correlation to C-27 (*δ* 64.4, *^3^J*), and the methane proton H-25 (*δ* 1.41) showed a COSY correlation with H-24 (*δ* 2.15), H-26 (*δ* 0.74) and H-27 (*δ* 3.16, 3.32), confirming the position of hydroxyl group. The configuration of C-20, C-24 and C-25 can not be defined only by NOESY experiment. However, there is only one single epimer which was isolated up to now. The stereochemistry of **1** at the chiral centers C-3, C-5, C-6, C-18, C-19 and C-17 were confirmed as the same as **5** on the basis of comparison of the NMR spectral data of **1** with those of compounds containing analogous side chain [11,12,13], and were further confirmed by NOESY spectral data. Accordingly, the structure of **1** was determined to be ergosta-7,22-diene-3β,5α,6β,27-tetraol and named as ananstrep A.

**Figure 3:**
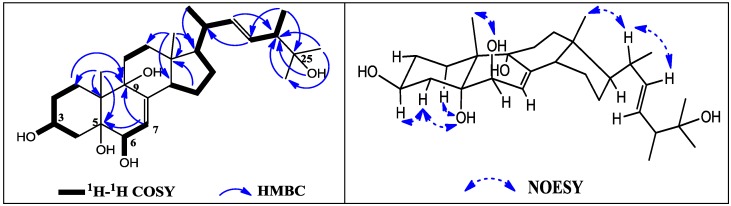
The key HMBC, H^1^–H^1^ COSY (left) and NOESY (right) correlations of **2**.

Compound **2** was also obtained in needle crystal. Its molecular formula, C_28_H_46_O_5_, was deduced from the [M + Na] ^+^ ion at *m/z* 485.3239 (C_28_H_46_NaO_5_, calcd. 485.3237) in HR-ESI-MS. Analysis of NMR spectra established that **2** was a polyhydroxylated 24-methylcholest-type sterol, similar to a previously isolated known compound **7** [14,15]. Actually, its ^1^H NMR spectrum showed four methyl singlets at *δ* 0.54 (Me-18), 0.96 (Me-19), 0.97 (Me-26) and 1.02 (Me-27), two methyl doublets at *δ* 0.98 (Me-21, *J* = 6.1 Hz), 0.90 (Me-28, *J* = 6.8 Hz), three olefinic protons at *δ* 5.12 (H-7, d, *J* = 4.5 Hz), 5.19 (H-22, dd, *J* = 15.1, 7.8 Hz) and 5.35 (H-23, dd, *J* = 15.1, 7.0 Hz). In the ^13^C and DEPT NMR spectra, there are 28 skeleton carbons including two double bonds at *δ* 120.3 (C-7), 141.1 (C-8), 135.8 (C-22) and 130.5 (C-23), four oxygenated carbons at *δ* 65.8 (C-3), 73.7 (C-5), 71.6 (C-6) and 77.3 (C-9), and an additional oxygenated quaternary carbon at *δ* 70.7 (C-25). Comparison of its NMR spectral data of **2** with those of **7** [14,15] revealed that the only obvious difference between them was the side chain, that is, one methine of **7** was oxygenated into a quaternary carbon in **2**. Positions of hydroxyl group at C-25 were confirmed by the long-range correlations (Figure 3). A hydroxyl singlet at *δ* 4.08 (27-OH) showed an HMBC correlation to C-24 (*δ* 47.2, *^3^J*), a methyl singlet at *δ* 0.97 (Me-26) displayed HMBC correlations to C-24 (*δ* 47.2, *^3^J*) and C-27 (*δ* 28.3, *^3^J*), while a methyl doublet at *δ* 0.90 (Me-28) showed HMBC correlations to C-23 (*δ* 130.5, *^3^J*) and C-25 (*δ* 70.7, *^3^J*). All the NMR spectral data suggested that the additional hydroxyl group at *δ* 4.08 (1H, s) attached to C-25 (*δ* 70.7). On the basis of the above data, the stereochemistry of **2** can be considered as the same as compound **7**. Thus, **2** was established as ergosta-7,22-diene-3β,5α,6β,9α,25-pentol and named as ananstrep B.

Compound **3** was isolated as white powder. The HR-ESI-MS spectrum of **3** showed a quasi-molecular [M + Na]^+^ ion at *m/z* 451.3218 (calcd. 451.3183), ascribe to the molecular formula C_28_H_44_NaO_3_, its degree of unsaturation was seven. The ^1^H NMR spectrum displayed characteristic signals of ergosterol for two methyl singlet groups at *δ* 0.57 (Me-18) and 1.17 (Me-19), four methine doublets at *δ* 0.98 (Me-21, d, *J* = 6.3 Hz), 0.79 (Me-26, d, *J* = 4.1 Hz), 0.80 (Me-27, d, *J* = 4.1 Hz) and 0.88 (Me-28, d, *J* = 6.8 Hz), and only two olefinic protons at *δ* 5.18 (H-22, dd, *J* = 15.1, 8.0 Hz) and 5.20 (H-23, dd, *J* = 15.1, 7.2 Hz). The ^13^C and DEPT spectra showed 28 signals including three oxygenated methane at *δ* 67.0 (C-3), 60.0 (C-6) and 65.0 (C-7), one oxygenated quaternary carbons at *δ* 62.6 (C-5), and four characteristic downfield signals at *δ* 126.2 (C-8), 134.4 (C-9), 131.3 (C-23) and 135.5 (C-22). All of the above data indicated that **3** might be an ergosterol epoxide.

**Figure 4:**
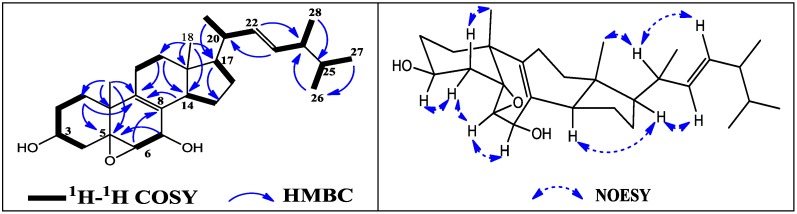
The key HMBC, H^1^-H^1^ COSY (**left**) and NOESY (**right**) correlations of **3**.

Interpretation of the COSY spectrum of **3** gave several spin systems corresponding to substructures as shown with bold lines in Figure 4. In the HMBC spectrum, one methyl singlet at *δ* 0.57 (Me-18) displayed four HMBC correlations to C-12 (*δ* 36.1, ^3^*J*), C-13 (*δ* 41.5, ^2^*J*), C-14 (*δ* 51.3, ^3^*J*), C-17 (*δ* 53.8, ^3^*J*), the other methyl singlet at *δ* 1.17 (Me-19) displayed four HMBC correlations to C-1 (*δ* 30.6, ^3^*J*), C-5 (*δ* 62.6, ^3^*J*), C-9 (*δ* 134.4, ^3^*J*) and C-10 (*δ* 37.3, ^2^*J*), taken together with correlations from H-7 (*δ* 4.10) to C-5 (*δ* 62.6), from H-6 (*δ* 2.87) to C-8 (*δ* 126.2), from H-12 (*δ* 1.88, 1.34) to C-9 (*δ* 134.4) and C-14 (*δ* 51.3), and from H-14 (*δ* 2.00) to C-9 (*δ* 134.4), indicated that **3** was a 5,6-epoxy-8(9),22-diene-3,7-diol-ergosterol, whose plane structure was the same as that of a previously isolated compound **8**.

Compared NMR spectra data of **3** in CDCl_3_ with those of **8** [16,17], there were visible changes at C-5 (*δ* 63.4), C-6 (*δ* 60.3), C-7 (*δ* 67.0), C-8 (*δ* 126.7), C-9 (*δ* 137.2), C-14 (*δ* 51.3) and C-17 (*δ* 54.5), which further confirmed the difference of the relative structure of **3** from that of **8**. The relative stereochemistry of this sterol nucleus was deduced by NOESY spectra data in CDCl_3_, which demonstrated key correlations depicted in Figure 3. NOE correlations from He-4 (*δ* 1.45) with H-3 (*δ* 3.91) and H-6 (*δ* 3.12), from H-6 (*δ* 3.12) with H-7 (*δ* 4.36) were observed, while Ha-4 (*δ* 2.20) showed a strong NOE correlation with Me-19 (*δ* 1.25), thus indicating the β-orientation at C-5, C-6 and C-7 of **3**, which was the most different part from those of **8**. The configuration of Me-18 and stereochemistry of the side chain of **3** can be considered as the same as **8** [16,17] because of the identical ^1^H and ^13^C NMR spectra data, which were also deduced by NOESY experiments. Therefore, the structure of **3** was determined as 5β,6β-epoxy-ergosta-7,22-diene-3β,7β-diol and named as ananstrep C. It is worthy to note that compound **3** possess a rare β-orientation at C-5 and C-6, which are commonly *α*-orientation in this kind of compounds, just like those in compound **8**. However, what is most interesting is that compounds **3** and **8** are two compounds from mutually adjacent peaks in HPLC profiles of a fraction, and both of them showed better inhibitory activity to cancer cell lines. The full ^1^H and ^13^C assignments (Table 1 and Table 2) were made based on the 2D NMR spectroscopic interpretations.

Compounds **4**–**13** were characterized as ergosterols by analysis of MS and NMR data and comparison with those in literature. They were identified to be ergosta-7,22-diene-3β,5α,6β,25-tetraol (**4**) [13], ergosta-7,22-diene-3β,5α,6β-triol (**5**) [11,12], ergosta-7,22-diene-3β,5α,6α-triol (**6**) [18], ergosta-7,22-diene-3β,5α,6β,9α-tetraol (**7**) [14,15], 5α,6α-epoxy-ergosta-8(9),22-diene-3β,7α-diol (**8**) [16,17], 5α,6α-epoxy-ergosta-8(14),22-diene-3β,7α-diol (**9**) [19], ergosta-8(9),22-diene-3β,5α,6β,7α-tetraol (**10**) [20], ergosta-8(14),22-diene-3β,5α,6β,7α-tetraol (**11**) [21], ergosta-5,7,22–triene-3β-ol (**12**) [22] and 5β,6β-epoxy-ergosta-8(14),22-diene-3β,7β-diol (**13**) [23]. Most of them were isolated from fungi and sponges earlier.

Why there are so many ergosterols isolated from the ethanol extract of mycelium of strain H41-59? As we all know, ergosterol is a chemical constituent mainly found in fungi, and there is no solid evidence to show that the actinomycete biosynthesizes ergosterol carbon skeleton. However, cholesterol oxidase (ChO), one of the key enzymes of microbial sterol metabolism, was usually observed among Gram-positive G + C-rich actinobacteria, e.g., *Streptomyces* [24,25], *Nocardia* [26], *Mycobacterium* [27], *Gordonia* [28], *Rhodococcus* [29], and some genes encoding different ChOs have been cloned from some actinomycete. Therefore, actinobacteria are capable of effecting diverse types of sterol transformation, such as hydroxylation, dehydrogenation, double bond isomerization and partial degradation of the side chain or sterol nucleus [30].

**Scheme 1:**
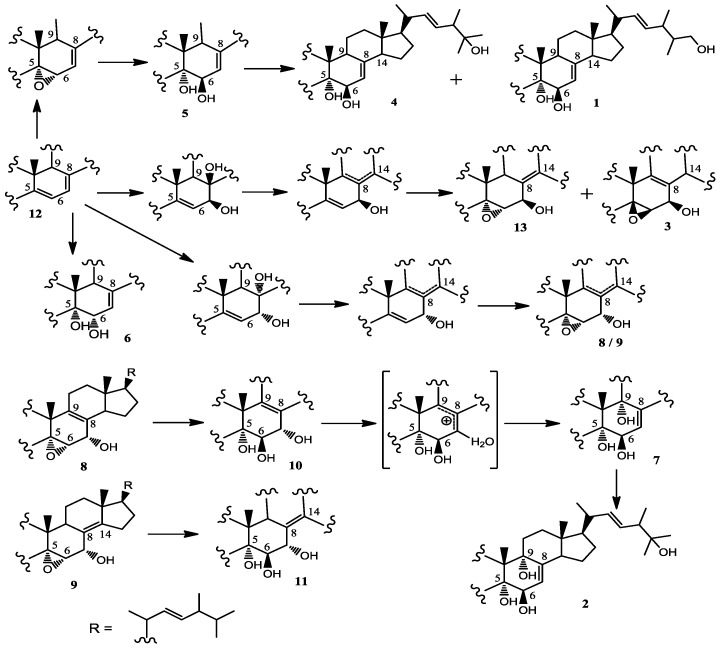
Plausible biosynthetic pathway of **1**–**13**.

Accordingly, it is not far-fetched to imagine that one or some known ergosterols originated in yeast powder, a nitrogen source of culture medium, might be transformed into other ergosterols including three new ones, and the plausible biotransformation was hypothesized as shown as Scheme 1 [31,32,33].

### 2.2. Biological Activity

The isolated metabolites **1**–**13** showed no bioactivity against *Candida albicans*, *Escherichia coli*, *Staphylococcus aureus*, *Bacillus* sp. and *Dickeya zeae* at 1 mg/mL, while the crude extract displayed a moderate activity against five test strains. The antibacterial activity of the extract may come from the unisolated minor components in the extract. The cytotoxicity against human breast adenocarcinoma cell line MCF-7, human glioblastoma cell line SF-268, and human lung cancer cell line NCI-H460 has also been investigated using an MTT assay. As is described in Table 3, all the isolated compounds showed to some extent cytotoxicity against three cancer cell lines. According to the cytotoxicity result, it is worthy to note that those △^8(9)^-sterols and△^8(14)^-sterols such as compounds **3,** **8**, **10** and **11** were likely to display better effect against three tumor cell lines. Among them, compound **3**, possessing a △^8(9)^ moiety, indicated much better effect against SF-268, MCF-7 and NCI-H460 with IC_50_ value of 13.0, 18.1 and 23.5 μg/mL, respectively.

## 3. Experimental Section

### 3.1. General Experimental Procedures

Melting points were recorded on an X-5 micro-MP apparatus (Huayan Corporation, Shanghai, China), uncorrected. UV spectra were determined on a JASCO V-550 UV/VIS spectrometer (JASCO Corporation, Tokyo, Japan). IR spectra were carried out with a Nicolet Impact 410-FTIR instrument (Thermo, San Jose, CA, USA) in KBr pellets. The optical rotations were measured with a JASCO digital polarimeter (JASCO Corporation, Tokyo, Japan). HR-ESI-MS were acquired on an Agilent 6210 LC/MSD TOF mass spectrometer (Agilent Technologies, Santa Clara, CA, USA). NMR spectrawere performed on Bruker AV-300 and AV-600 spectrometer (Bruker Instrument, Inc., Zurich, Switzerland), tetramethylsilane (TMS) was used as the internal standard for ^1^H NMR, and referencing to the NMR solvent used for ^13^C NMR. Chemical shifts were evaluated in *δ* (ppm). HPLC was performed on an Agilent 1200 HPLC system equipped with an diode array detector, using a column A (Ultimate XB-C18, 5 μM, 4.6 × 250 mm, Welch, Potamac, MA, USA) for analysis and a column B (Ultimate XB-C18, 5 μm, 10 × 250 mm, Welch, Potamac, MA, USA) for semi-preparative purification. Open column chromatography was performed on silica gel (300–400 mesh, Qingdao, Haiyang Chemical Group Corporation, Qingdao, China). Sephadex LH-20 (25–100 mm) was purchased from Pharmacia (Uppsala, Sweden). HSGF254 silica gel TLC plates (0.2 mm thickness, 200 × 200 mm, Qingdao Marine Chemical, City, China) were used as routine analysis of fractions. Strains *Candida albicans*, *Escherichia coli*, *Staphylococcus aureus*, *Bacillus* sp., and *Dickeya zeae* were obtained from the Institute of New Drug Research in our college.

### 3.2. Strain Isolation and Identification

The actinomycete strain H41-59 was isolated in 2001 from a marine sediment sample collected at the Zhapo mangrove site, on the Hailing island of Yangjiang, Guangdong province, China, and kept in a sandy soil tube and stored at about 4 °C refrigerator before use. Strain H41-59 was activated on Gauserime synthetic agar medium at 30 °C. Studies on its morphological, physiological and biochemical characteristic indicated that this strain belonged to the genus *Streptomyces*. Analysis of its 16S rDNA gene sequence let us identify this strain as *Streptomyces anandiidue* by its 99.9% similarity with the sequence of *S. anandii* deposited in GenBank under accession number GU350497.1. A voucher specimen of strain H41-59 was preserved in the Guangdong Key Laboratory of New Technique for Plant Protection, Institute of Plant Protection, Guangdong Academy of Agricultural Sciences, Guangzhou, China.

### 3.3. Fermentation and Extraction

Strain H41-59 was grown under shaking condition at 28 °C and 170 rpm for two days in four 1 L Erlenmeyer flasks containing 250 ml of the seed medium (composed of yeast powder 30 g, corn starch 30 g, crude salt 2.5 g, CaCO_3_ 1.5 g, KNO_3_ 1 g, MgSO_4_ 0.6 g, K_2_HPO_4_ 0.9 g, FeSO_4_ 0.02 g, H_2_O 1L, adjusting pH to 7.4 with NaOH solution), then inoculated into production medium (20 L) in a 30-L fermentor and incubated for five days at 28 °C. After subjecting to 20 L scale fermentation four times up to total volume of 80 L, the culture broth was centrifuged at 4416 g and then the mycelium was percolated using 95% ethanol (EtOH). After removal of EtOH with a rotatory evaporator under vacuum at 50 °C, the aqueous solution was diluted with distilled water, then added on macroporous resin HP2MGL column (Mitsubishi Chemical, Japan) to absorb secondary metabolites. The column was successively washed with water, 50% EtOH, and 95% EtOH. The 95% EtOH elution was concentrated using rotatory evaporator in vacuum and under 50 °C. Then, the residual material was suspended in water and extracted with ethyl acetate (EtOAc). Finally, the EtOAc solution was evaporated to give a residue (50 g).

### 3.4. Isolation and Purification

The EtOAc extract was dissolved in chloroform and loaded on the silica gel column (300–400 mesh, Qingdao, China) after filtration. A stepwise gradient elution of petroleum ether-EtOAc (10:0, 9:1, 8:2, 7:3, 6:4, 5:5, 4:6, 2:8 and 0:10 (*v/v*)) was used, and 20 fractions (Fr-1 to Fr-20) were obtained through TLC analysis and combination of fractions with same TLC pattern. Fr-12 was subsequently subjected to silica gel column chromatography (CC) eluting with a stepwise gradient of chloroform-methanol (MeOH) (20:1, 10:1 and 5:1(*v/v*)) to yield five subfractions (Fr-12-1 to Fr-12-5). Fr-12-4 was separated by semi-preparative HPLC eluting with 70% MeOH to yield **4** (9.4 mg, RT = 20.806 min). Fr-12-5 was further purified by extensive semi-preparative C-18 RP-HPLC using 90% MeOH to give compound **7** (10.5 mg, RT = 10.138 min), **10** (1.4 mg, RT = 14.399 min), **11** (2.1 mg, RT = 13.172 min), and a subfraction Fr-12-5-1 (75 mg). Then, subfraction Fr-12-5-1 was purified by semi-preparative HPLC using 75% MeOH as an eluent to give compounds **1** (2.4 mg, RT = 22.674 min), **2** (43 mg, RT = 10.396 min); Fr-8 was performed on semi-preparative HPLC eluting with MeOH-H_2_O (50:50→100:0) to give compound **3** (13.2 mg, RT = 34.238 min), **8** (12.9 mg, RT = 34.585 min), and **6** (3.1 mg, RT = 36.255 min); compound **9** (1.5 mg, RT = 42.333 min) and **13** (10 mg, RT = 41.338 min) was semi-prepared by HPLC using 85% MeOH from Fr-6 and Fr-7, respectively. All preparative HPLC methods were performed in the flow rate of 3 ml/min and detected at UV 210 nm. Compounds **5** (20.6 mg) and **12** (30.1 mg) were obtained by crystallization as colorless needle crystal from Fr-12 and Fr-4, respectively.

**Ananstreps A (1)**: Colorless needle crystal; M.P = 252–253 °C; ${\left[\alpha \right]}_{D}^{23}$−23.76° (*c* 0.25, MeOH); UV (MeOH) λ_max_ (logε): 204.6 (3.88) nm; IR (KBr) *v*_max_: 3436, 2954, 2870, 1638, 1455, 1383, 1192, 1110, 620 cm^−1^; ^1^H NMR (DMSO-*d*_6_, 300 MHz) and ^13^C NMR (DMSO-*d*_6_, 75 MHz), see Table 1 and Table 2; HR-ESI-MS: *m/z* 469.3293 ([M + Na]^+^, C_28_H_46_NaO_4_, calcd. 469.3288).

**Ananstreps B (2)**: Colorless needle crystal; M.P = 155–156 °C; ${\left[\alpha \right]}_{D}^{23}$−4.84° (*c* 0.25, MeOH); UV (MeOH) λ_max_ (logε): 205 (3.93) nm; IR (KBr) *v*_max_: 3420, 2956, 2871, 1646, 1455, 1377, 1114, 620 cm^−1^; ^1^H NMR (DMSO-*d*_6_, 300 MHz) and ^13^C NMR (DMSO-*d*_6_, 75 MHz), see Table 1 and Table 2; HR-ESI-MS: *m/z* 485.3239 ([M + Na]^+^, C_28_H_46_NaO_5_, calcd. 485.3237).

**Ananstreps C (3)**: Colorless needle crystal; M.P = 120–121 °C; ${\left[\alpha \right]}_{D}^{23}$−2.63° (*c* 0.25, MeOH); UV (MeOH) λ_max_ (logε): 206.4 (3.90) nm; IR (KBr) *v*_max_: 3436, 2956, 2870, 1641, 1458, 1371, 1193, 1110, 620 cm^−1^; ^1^H NMR (DMSO-*d*_6_, 300 MHz) and ^13^C NMR (DMSO-*d*_6_, 75 MHz), see Table 1 and Table 2; HR-ESI-MS: *m/z* 451.3218 ([M + Na]^+^, C_28_H_44_NaO_3_, calcd. 451.3183).

### 3.5. Biological Activities

#### 3.5.1. Antimicrobial Activity

Antimicrobial assays were assessed with an agar disk diffusion assay. The test samples were prepared in MeOH solutions at a concentration of 1 mg/mL, the filter paper disks (6 mm) were soaked in the solutions. Then, the disks were allowed to air dry at room temperature under sterile condition, next placed on inoculated agar plates and incubated for 12 h at 30 °C for fungal and 48 h at 35 °C for bacteria. The mycelium extract of *Streptomyces anandii* and the corresponding compounds **1**–**13** were examined against *Candida albicans*, *Escherichia coli*, *Staphylococcus aureus*, *Bacillus* sp. and *Dickeya zeae*.

#### 3.5.2. Cytotoxicity Assay

Three cancer cell lines MCF-7, SF-268, and NCI-H460 were obtained from School of Medicine, Jinan University (Guangzhou, China), and were cultured in RPMI 1640 medium (Life Technologies, Grand Island, NY, USA) supplemented with 10% fetal bovine serum (FBS, Gibco, Carlesbad, CA, USA), 100 U/mL penicillin and 100 μg/mL streptomycin (Invitrogen, Carlsbad, CA, USA). Cells were cultured at 37 °C in a humidified atmosphere of 5% CO_2_. The cytotoxicity of isolated compounds against three cancer cell lines was evaluated by an MTT assay with *cis*-dichlorodiamine platinum as a positive control. Seven concentrations (1.56, 3.13, 6.25, 12.5, 25, 50 and 100 μg/mL) of compounds **1**–**13** were set for the test. Briefly, the cells were seeded at 8 × 10^4^ /mL in 96-well plates overnight. When cells fusion reached 80% of the bottom, thirteen compounds and *cis*-dichlorodiamine platinum at different concentrations were added, and cultured for 48 h. Subsequently, MTT dye (dissolved in deionized water) was added to the 96-well plates. Following this, the 96-well plates were incubated at 37 °C for another 4 h. The liquid supernatant were removed softly and DMSO were added (100 μL per well). The absorbance at 570 nm was monitored using a microplate reader (Bio-Rad, Hercules, CA, USA). The concentrations required to inhibit cell growth by 50% (IC_50_) were calculated using Origin 8 software (OriginLab, Northampton, MA, USA).

## 4. Conclusions

Three new ergosterols along with ten known ones (**4**–**13**) were isolated from the culture broth of *Sterptomyces* sp. H41-59, which was isolated from sea mud at mangrove district and identified as *Streptomyces anandii* on the basis of 16S rDNA gene sequence analysis. Structures of ananstreps A–C (**1**–**3**) were elucidated on the basis of extensive analysis of spectroscopic data, including HR-ESI-MS, and NMR. The cytotoxicities of all isolated sterols were tested by the MTT method. Ananstreps C (**3**) indicated moderate cytotoxic activity against MCF-7, SF-268, and NCI-H460 cell lines with IC_50_ values in a range from 13.0 to 27.8 μg/mL.

## Figures and Tables

**Table 1:** ^1^H NMR spectral data of new compounds **1**–**3** (300 MHz for ^1^H, *J* in Hz).

No.	1^a^	2^a^	3^a^	3^b^
1	1.30, m	1.15, m	1.61, m	1.74, m
	1.47, m	2.05, m		
2	1.24, m	1.31, m	1.45, m	1.62, m
	1.60, m	1.67, m	1.75, m	1.95, m
3	3.76, m	3.76, m	3.53, m	3.91, m
4	1.49, m	1.47, m	1.20, m	1.45, m
	1.89, m	1.91, m	1.99, m	2.20, m
5	-	-	-	-
6	3.37, m	3.50, m	2.87, d, 2.7	3.12, d, 2.7
7	5.08,m	5.12, d, 4.5	4.10, brs	4.36, d, 2.2
8	-	-	-	-
9	1.94, m	-	-	-
10	-	-	-	-
11	1.24, m	1.15, m	1.97, m	1.66, m
	1.66, m	2.05, m		2.07, m
12	1.25, m	1.51, m	1.34, m	1.45, m
	1.96, m	1.68, m	1.88, m	1.95, m
13	-	-	-	-
14	1.80, m	2.39, m	2.00, m	2.23, m
15	1.36, m	1.45, m	1.23, m	1.66, m
	1.48, m		1.69, m	2.07, m
16	1.24, m	1.25, m	1.25, m	1.30, m
	1.66, m	1.68, m	1.63, m	1.80, m
17	1.25, m	1.28, m	1.14, m	1.19, m
18	0.54, s	0.54, s	0.57, s	0.61, s
19	0.91, s	0.96, s	1.17, s	1.25, s
20	2.00, m	1.98, m	2.00, m	2.02, m
21	0.99, d, 6.6	0.98, d, 6.1	0.98, d, 6.3	0.99, d, 6.6
22	5.19, dd, 15.4, 7.9	5.19, dd, 15.1, 7.8	5.18, dd, 15.1, 8.0	5.16, dd, 15.2, 7.6
23	5.21, dd, 15.4, 7.1	5.35, dd, 15.1, 7.0	5.20, dd, 15.1, 7.2	5.18, dd, 15.2, 6.9
24	2.15, m	1.98, m	1.84, m	1.84, m
25	1.41, m		1.45, m	1.43, m
26	0.74, d, 6.9	0.97, s	0.79, d, 4.1	0.81, d, 4.5
27	3.16, m	1.02, s	0.80, d, 4.1	0.89, d, 4.5
	3.32, m			
28	0.92, d, 6.8	0.90, d, 6.8	0.88, d, 6.8	0.89, d, 6.8
3-OH	4.21, d, 5.4	4.33, d, 3.6	4.68, brs	
5-OH	3.58, s	5.00, s		
6-OH	4.49, d, 5.6	4.76, d, 5.5		
7-OH			4.76, brs	
9-OH		5.19, s		
25-OH		4.08, s		
27-OH	4.30, m			

^a^ Measured in DMSO-*d*_6_; ^b^ Measured in CDCl_3_.

**Table 2:** ^13^C NMR spectral data of new compounds **1**–**3** (75 MHz for ^13^C).

No.	1^a^	2^a^	3^a^	3^b^
1	32.5	26.9	30.6	31.0
2	31.2	31.0	30.8	31.0
3	66.0	65.8	67.0	68.8
4	40.2	40.2	39.0	39.2
5	74.5	73.7	62.6	63.4
6	72.1	71.6	60.0	60.3
7	119.5	120.3	65.0	67.0
8	139.7	141.1	126.2	126.7
9	42.3	77.3	134.4	137.2
10	36.6	39.6	37.3	38.0
11	27.8	26.9	22.4	23.2
12	38.9	35.0	36.1	36.3
13	43.0	43.3	41.5	42.1
14	54.2	50.1	51.3	51.3
15	22.6	22.6	29.1	23.1
16	27.8	27.8	22.1	29.4
17	55.3	55.5	53.8	54.5
18	12.1	11.7	11.3	11.7
19	17.7	21.4	22.5	23.8
20	40.1	40.1	40.0	40.7
21	21.0	20.9	20.8	21.2
22	135.7	135.8	135.5	135.0
23	130.3	130.5	131.3	132.3
24	36.9	47.2	42.1	43.0
25	40.5	70.7	32.5	33.3
26	13.1	26.0	19.5	19.9
27	64.4	28.3	19.8	20.2
28	18.3	15.0	17.4	17.8

^a^ Measured in DMSO-*d*_6_; ^b^ Measured in CDCl_3_.

**Table 3:** Cytotoxic activities of isolated compounds **1**–**13** (IC_50_: μg/mL).

Compounds	MCF-7	SF-268	NCI-H460	Compounds	MCF-7	SF-268	NCI-H460
**1**	>50	>50	>50	**7**	>50	>50	>50
**2**	>50	>50	>50	**8**	24.3	15.5	19.8
**3**	18.1	13.0	23.5	**9**	>50	>50	>50
**4**	>50	>50	>50	**10**	17.3	27.8	23.7
**5**	>50	>50	>50	**11**	27.4	25.1	23.7
**6**	>50	>50	>50	**12**	>50	>50	>50
**Positive control**	4.0	41.0	25.1	**13**	>50	>50	>50

Concentration range: 1.56–100 μg /mL; IC_50_: half maximal inhibitory concentration.

## Supplementary Materials

The following are available online at http://www.mdpi.com/1660-3397/14/5/84/s1, NMR, HR-ESI-MS, IR and UV spectra of compounds **1**–**3** as well as other supporting data.

## Abbreviations

HPLC	High performance liquid chromatography
DEPT	Distortionless enhancement by polarization transfer
COSY	Two dimensional ^1^H correlation
HSQC	^1^H-detected heteronuclear single-quantum coherence
HMBC	^1^H-detected heteronuclear multiple-bond correlation
NOESY	Nuclear overhauser effect spectroscopy
MTT	3-(4,5-dimethyl-2-thiazolyl)-2,5-diphenyl-2-H-tetrazolium bromide
