# Supplementary material for: Ergosterols from the Culture Broth of Marine Streptomyces anandii H41-59
Source: Mar Drugs. 2016 May 4;14(5):84. doi: 10.3390/md14050084 (PMC4882558; doi:10.3390/md14050084)
Supplement: Supplementary file 1 [file marinedrugs-14-00084-s001.pdf]

Supplementary Materials: Ergosterols from Culture Broth of a Marine Derived *Streptomyces* sp. H41-59

Yang-Mei Zhang, Hong-Yu Li, Chen Hu, Hui-Fan Sheng, Ying Zhang, Bi-Run Lin and Guang-Xiong Zhou

Compound 1

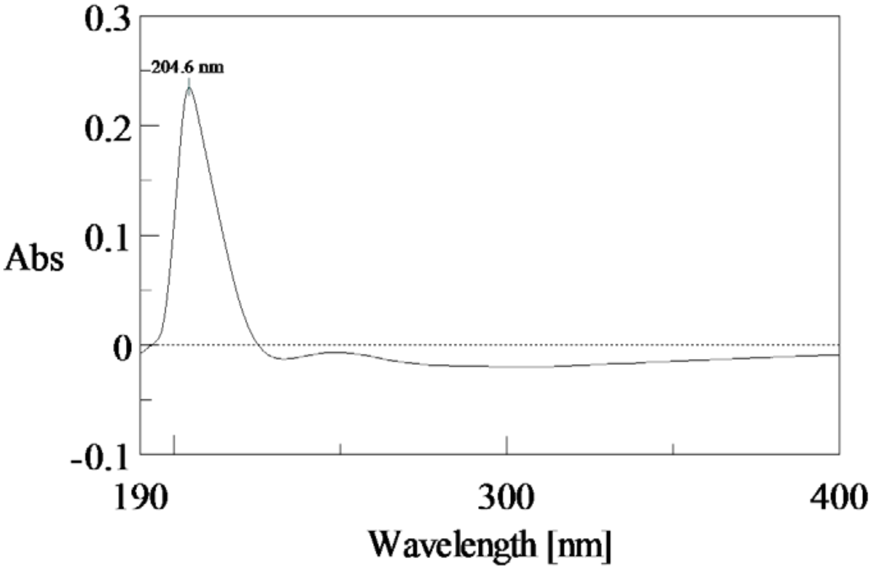

Figure S1. UV spectrum of compound **1** in MeOH.

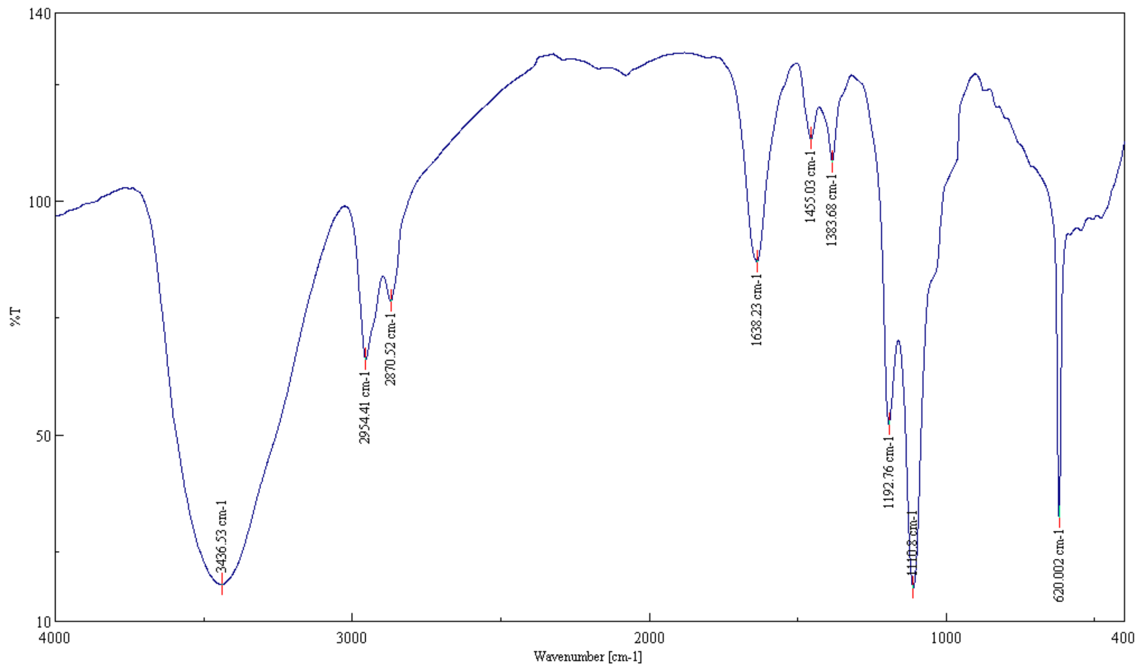

Figure S2. IR spectrum of compound **1**.

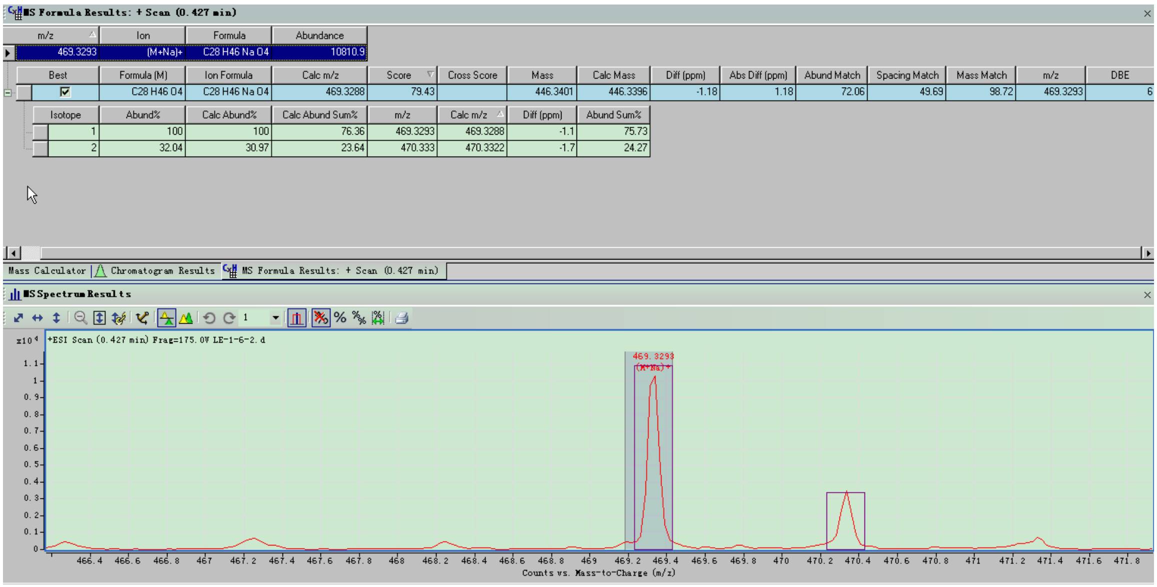

Figure S3. HR-TOF-MS of compound **1**.

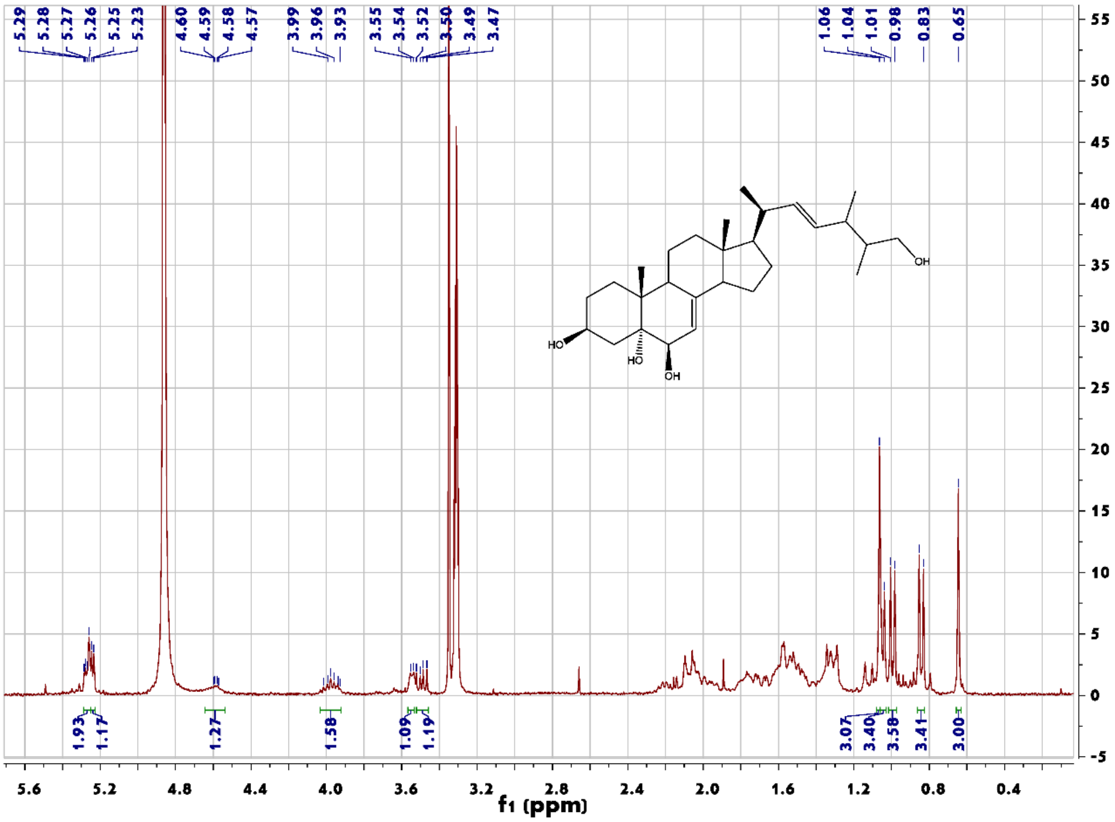

Figure S4. ^{1}H-NMR spectrum of compound **1** (300 MHz in MeOH).

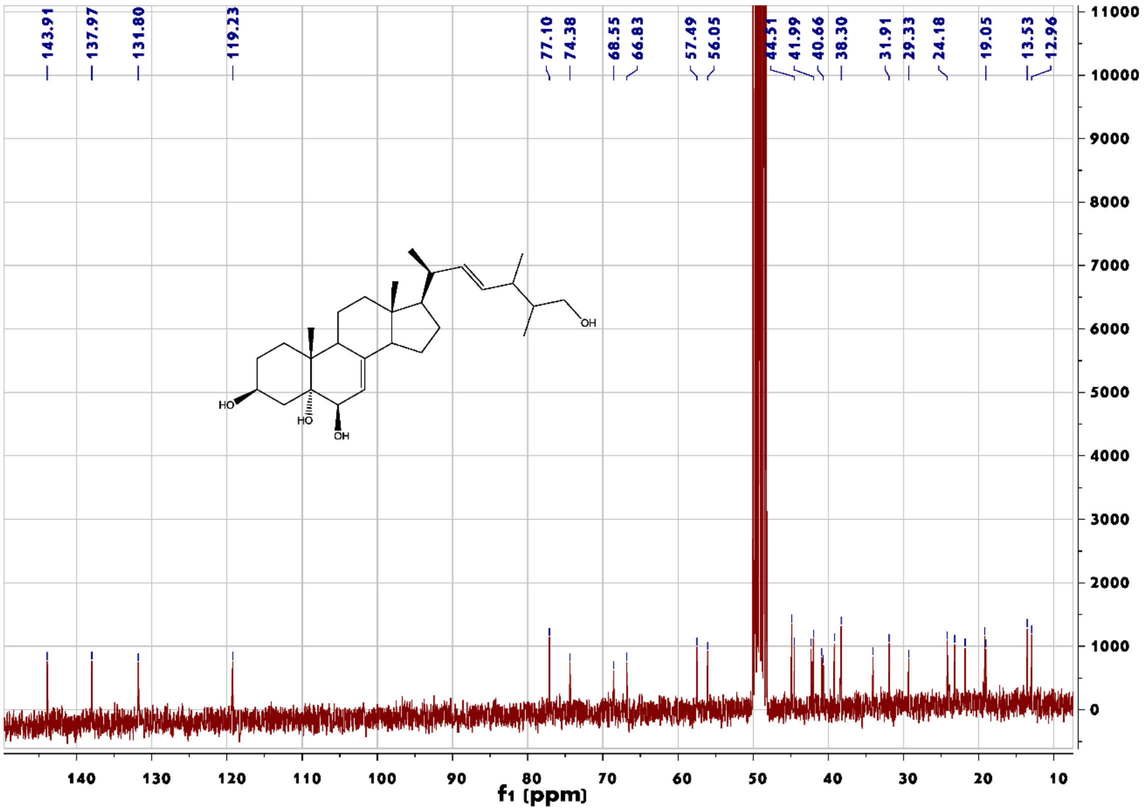

Figure S5. ^{13}C-NMR spectrum of compound **1** (75 MHz in MeOH).

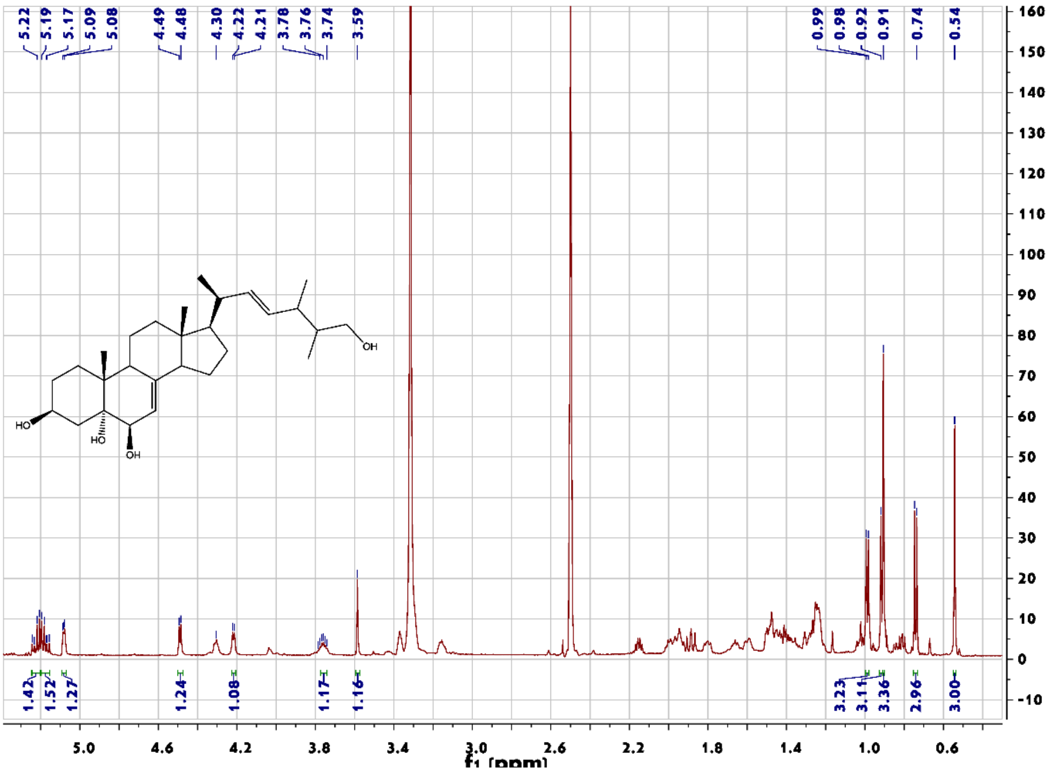

Figure S6. ^{1}H-NMR spectrum of compound **1** (300 MHz in DMSO-*d*6).

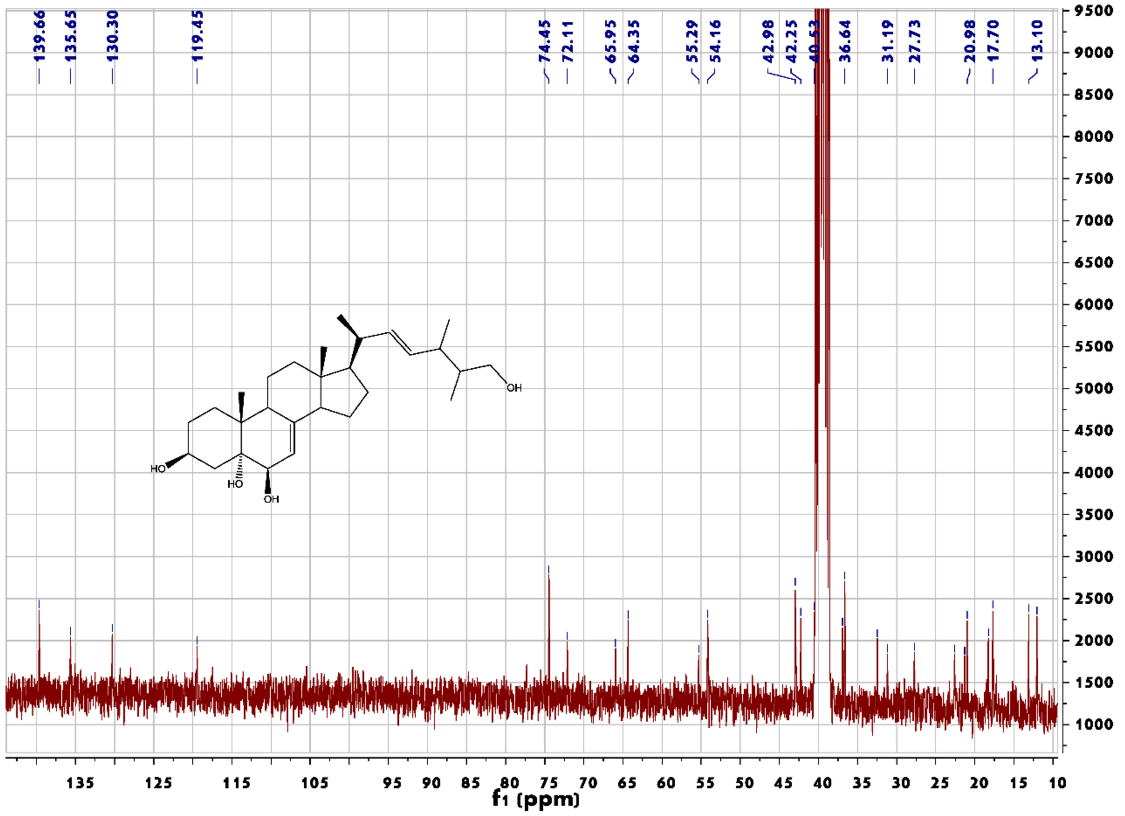

Figure S7. ^{13}C-NMR spectrum of compound **1** (75 MHz in DMSO-*d*6).

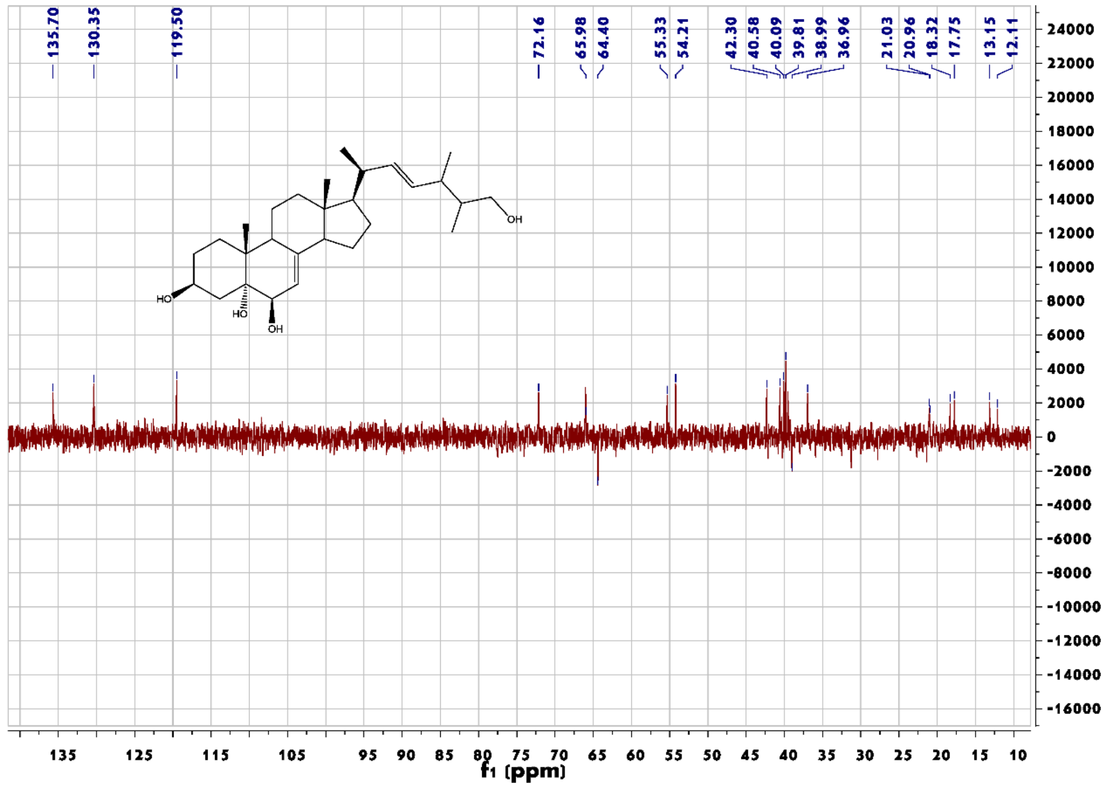

Figure S8. DEPT spectrum of compound **1** (75 MHz in DMSO-*d*6).

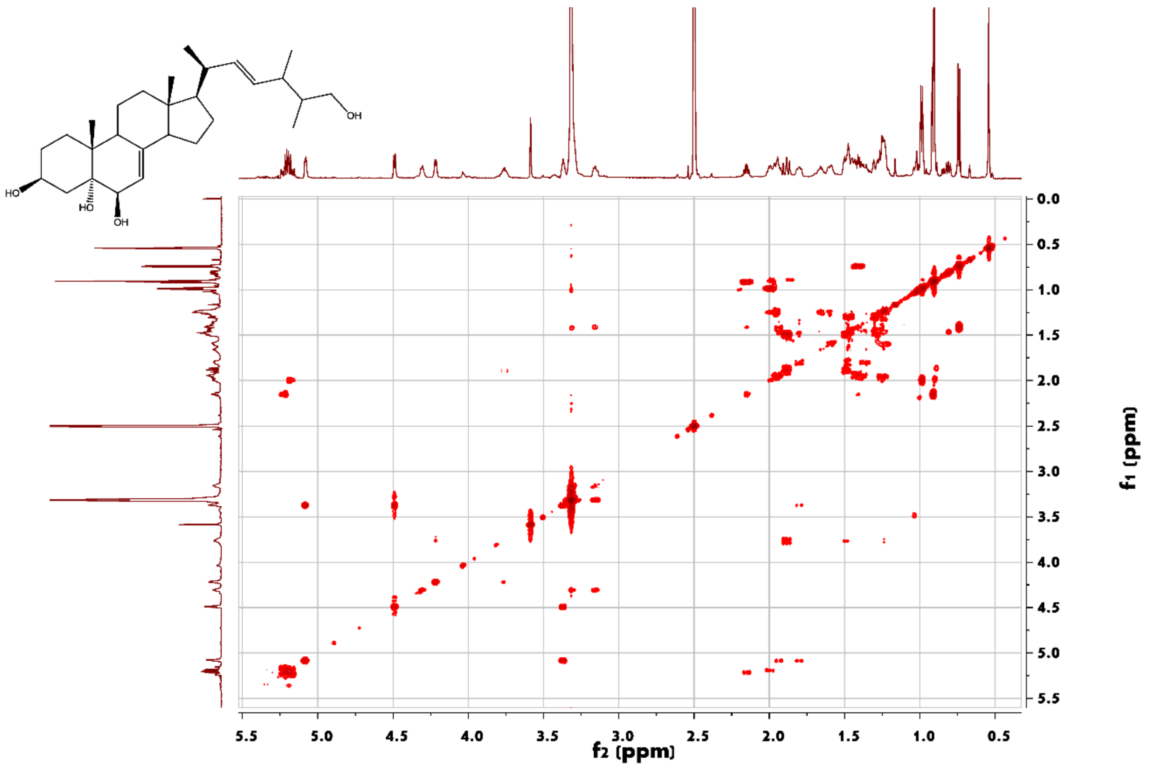

Figure S9. COSY spectrum of compound **1** (600 MHz in DMSO-*d*6).

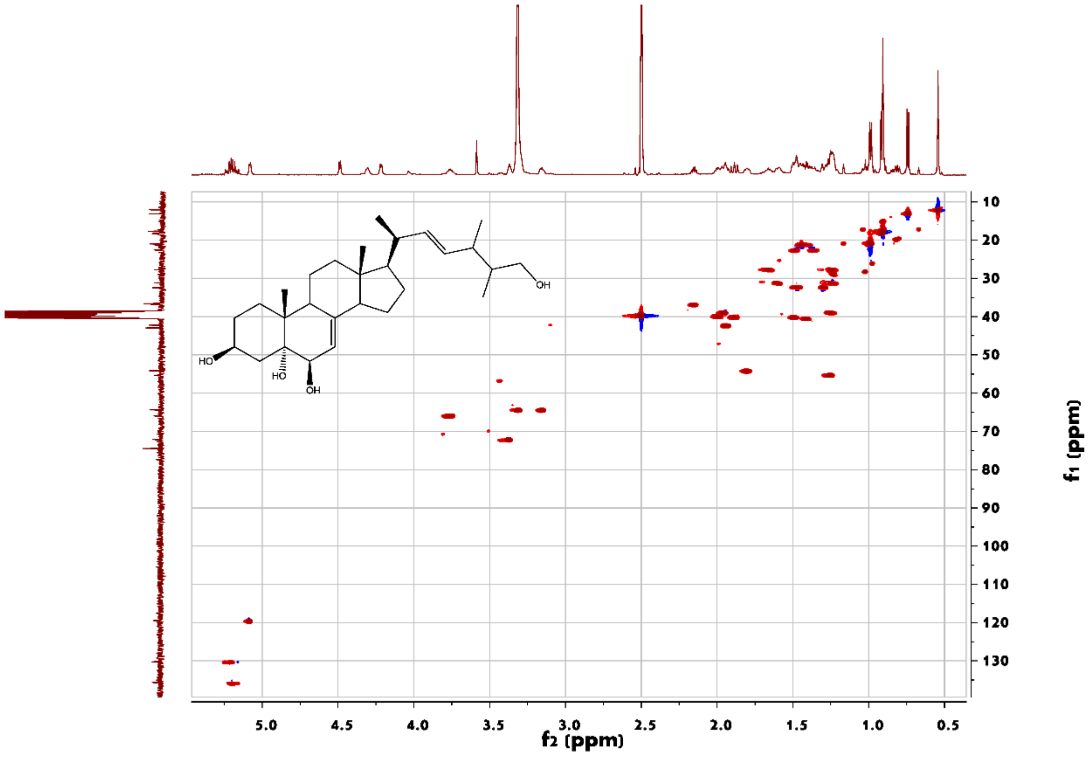

Figure S10. HMQC spectrum of compound **1** (600 MHz in DMSO-*d*6).

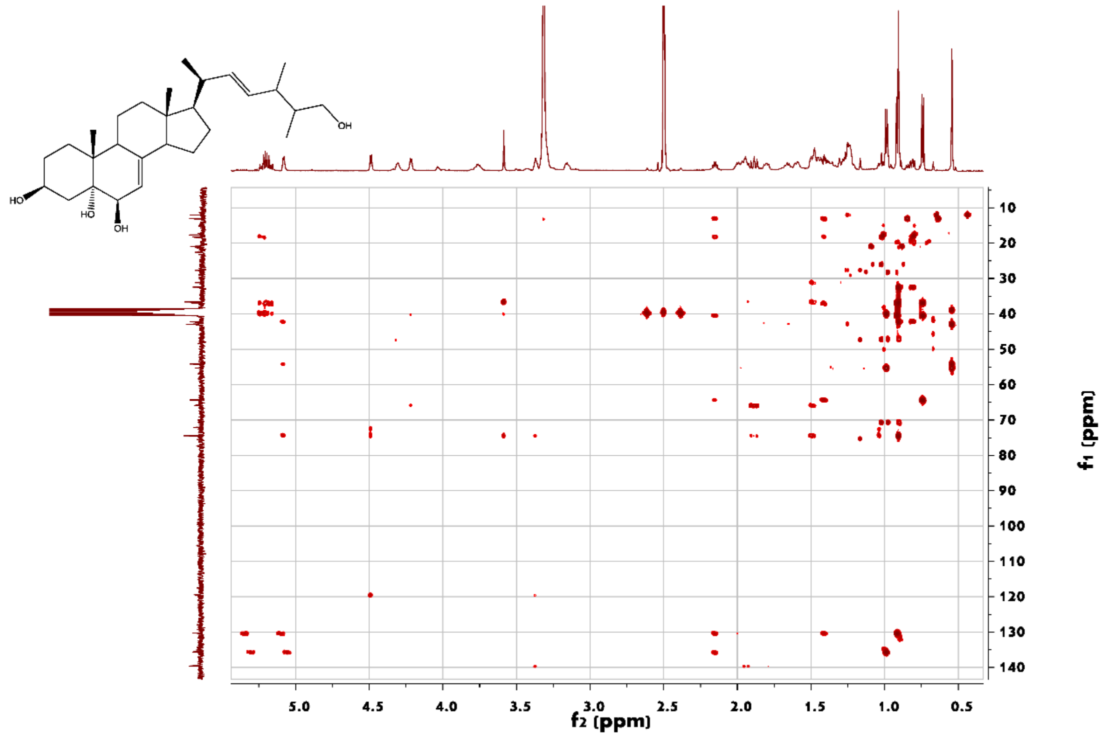

Figure S11. HMBC spectrum of compound **1** (600 MHz in DMSO-*d*6).

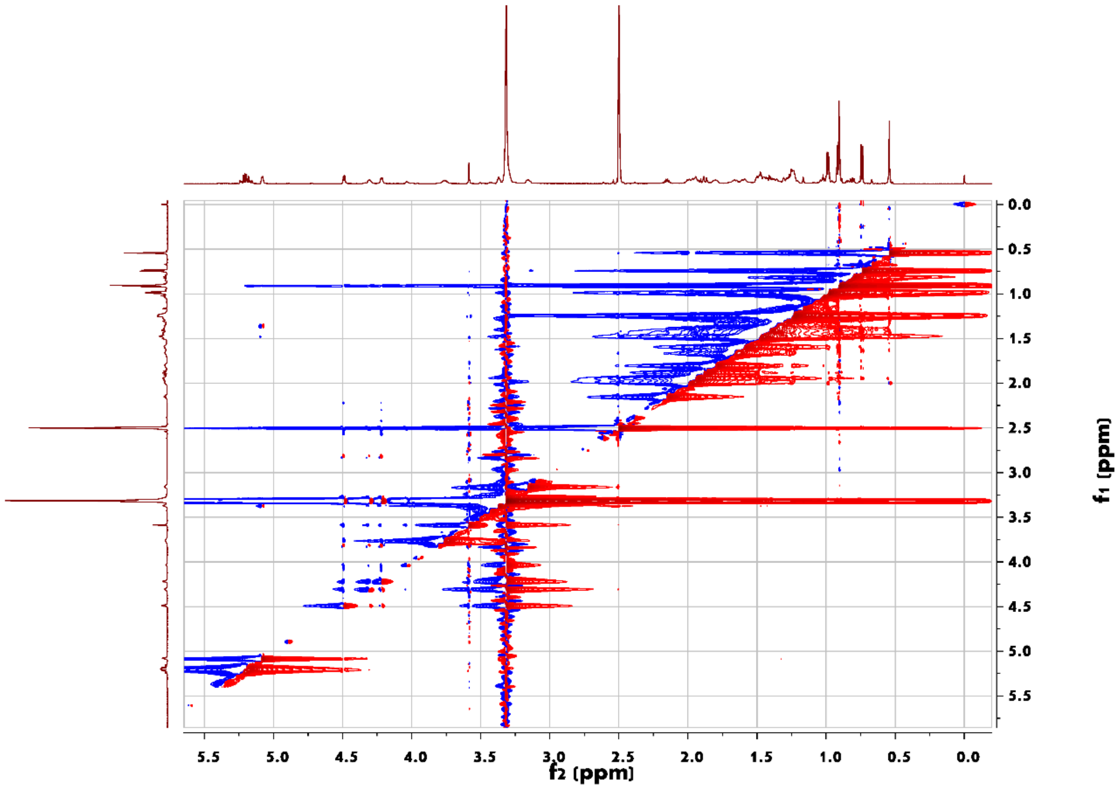

Figure S12. NOESY spectrum of compound **1** (600 MHz in DMSO-*d*6).

Compound 2

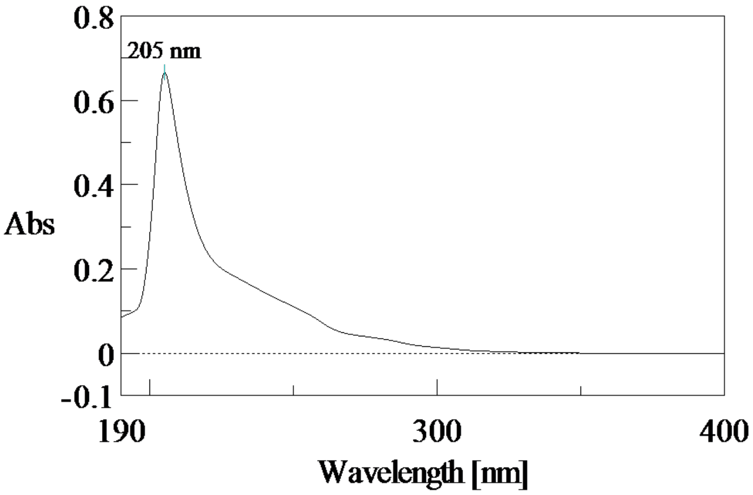

Figure S13. UV spectrum of compound **2** in MeOH.

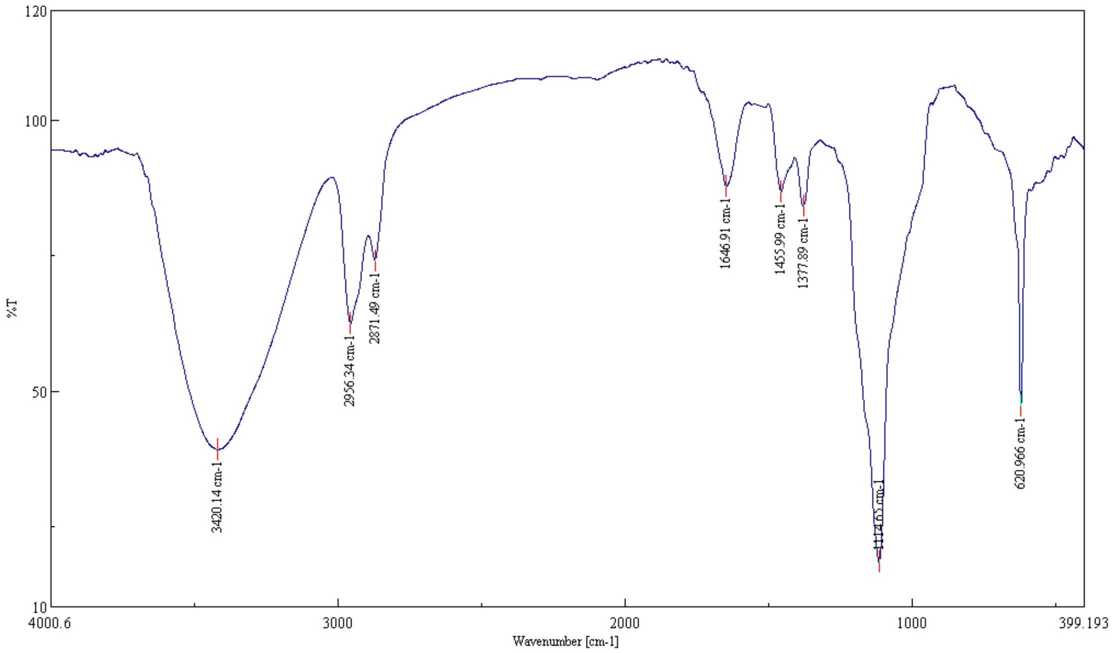

Figure S14. IR spectrum of compound **2**.

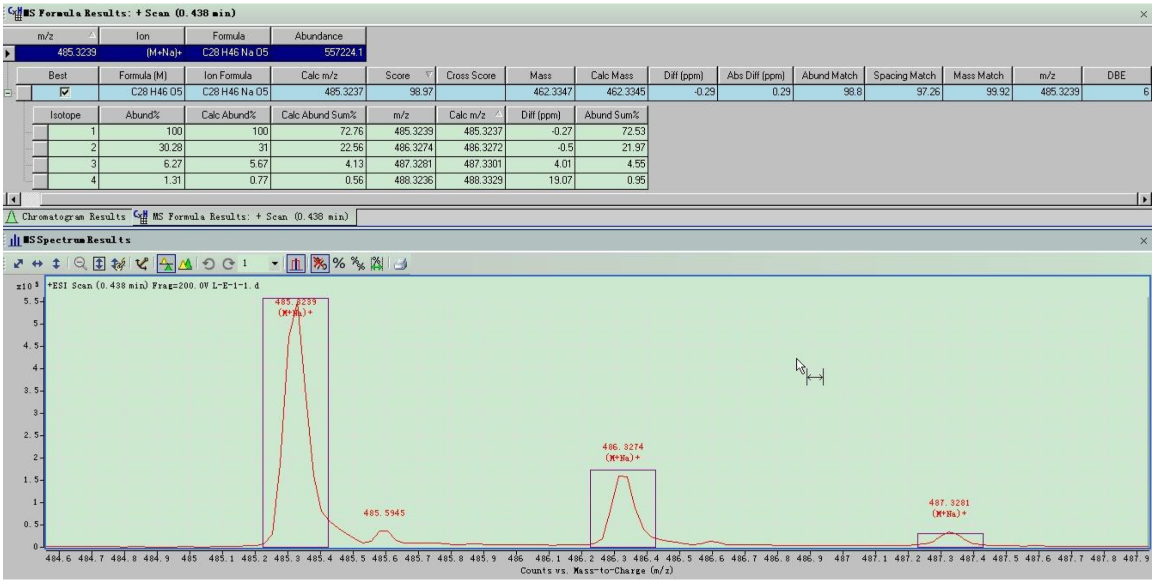

MS Formula Results: + Scan (0.438 min)

m/z	Ion	Formula	Abundance
485.3239	(M+Na)+	C28 H46 Na O5	557224.1

Best	Formula (M)	Ion Formula	Calc m/z	Score	Cross Score	Mass	Calc Mass	Diff (ppm)	Abs Diff (ppm)	Abund Match	Spacing Match	Mass Match	m/z	DBE
☑	C28 H46 O5	C28 H46 Na O5	485.3237	98.97		462.3347	462.3345	-0.29	0.29	98.8	97.26	99.92	485.3239	6

Isotope	Abund%	Calc Abund%	Calc Abund Sum%	m/z	Calc m/z	Diff (ppm)	Abund Sum%
1	100	100	72.76	485.3239	485.3237	-0.27	72.53
2	30.28	31	22.56	486.3274	486.3272	-0.5	21.97
3	6.27	5.67	4.13	487.3281	487.3301	4.01	4.55
4	1.31	0.77	0.56	488.3236	488.3329	19.07	0.95

Figure S15. HR-MS of compound **2**.

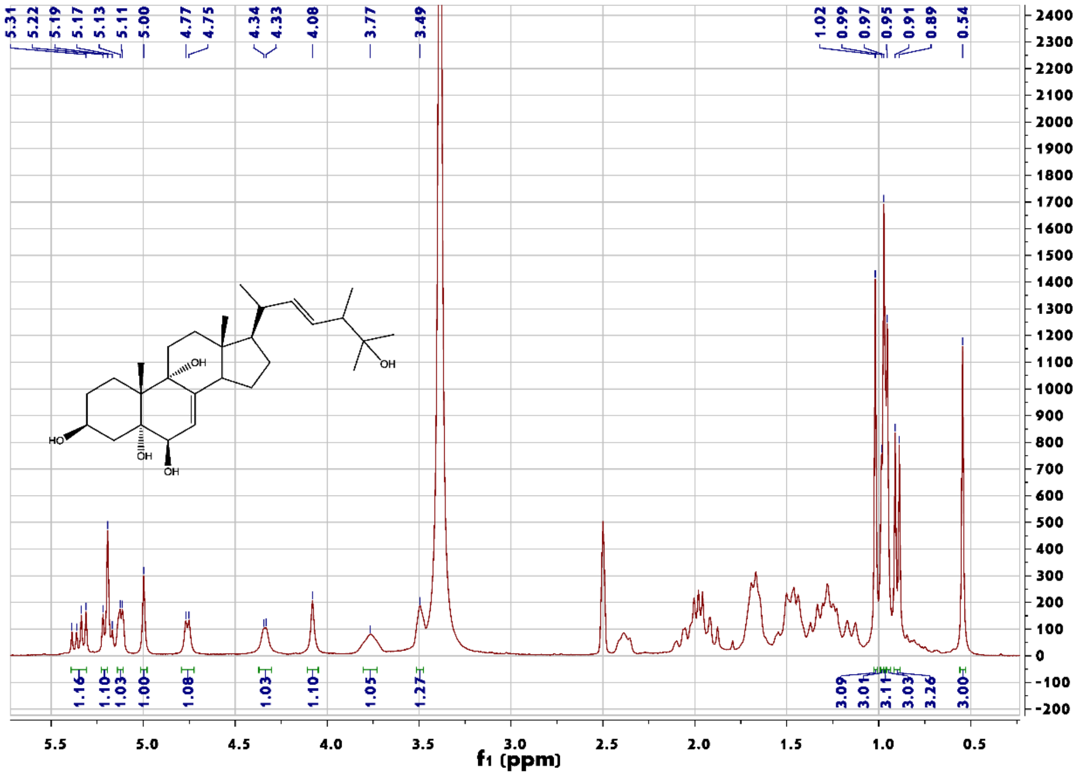

Figure S16. ^{1}H-NMR spectrum of compound **2** (300 MHz in DMSO-*d6*).

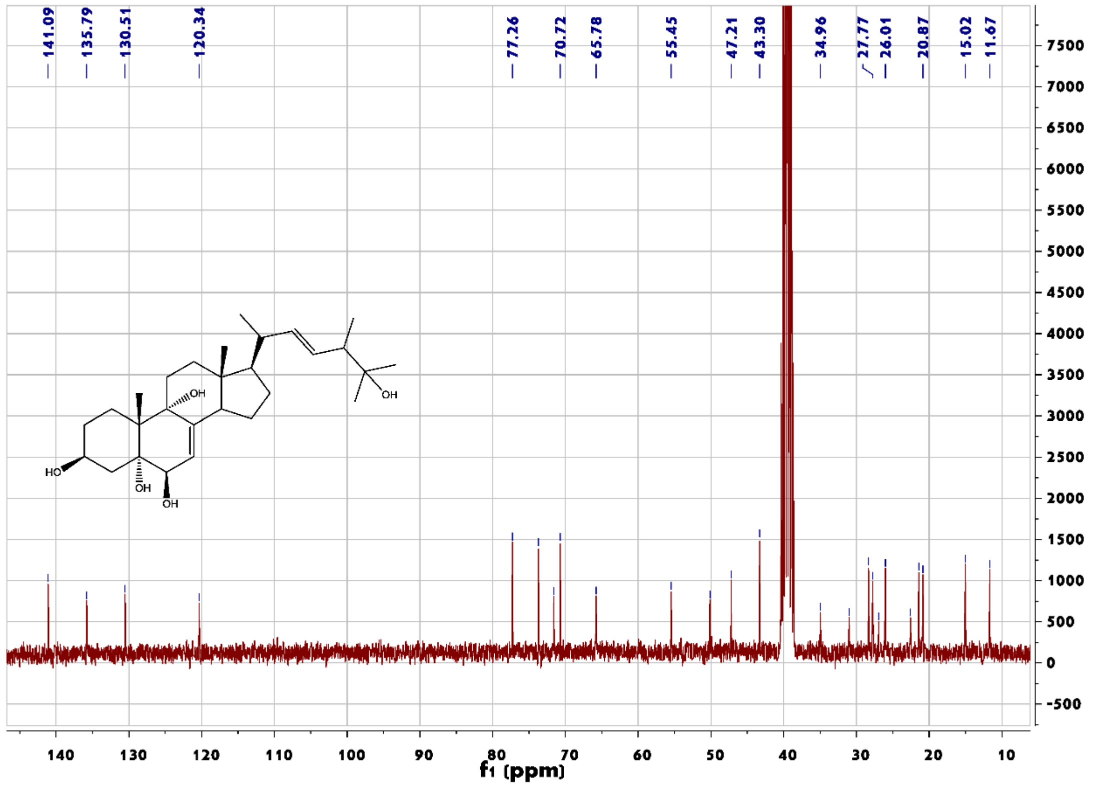

Figure S17. ^{13}C-NMR spectrum of compound **2** (75 MHz in DMSO-*d6*).

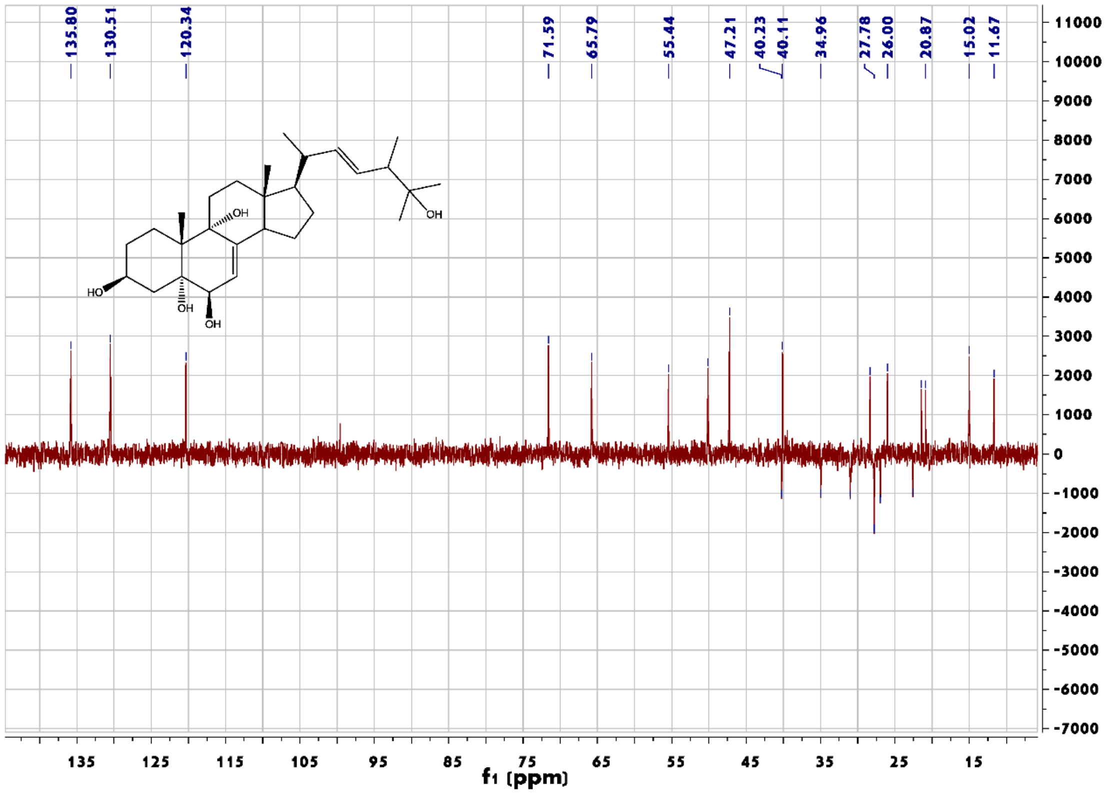

Figure S18. DEPT spectrum of compound **2** (75 MHz in DMSO-*d6*).

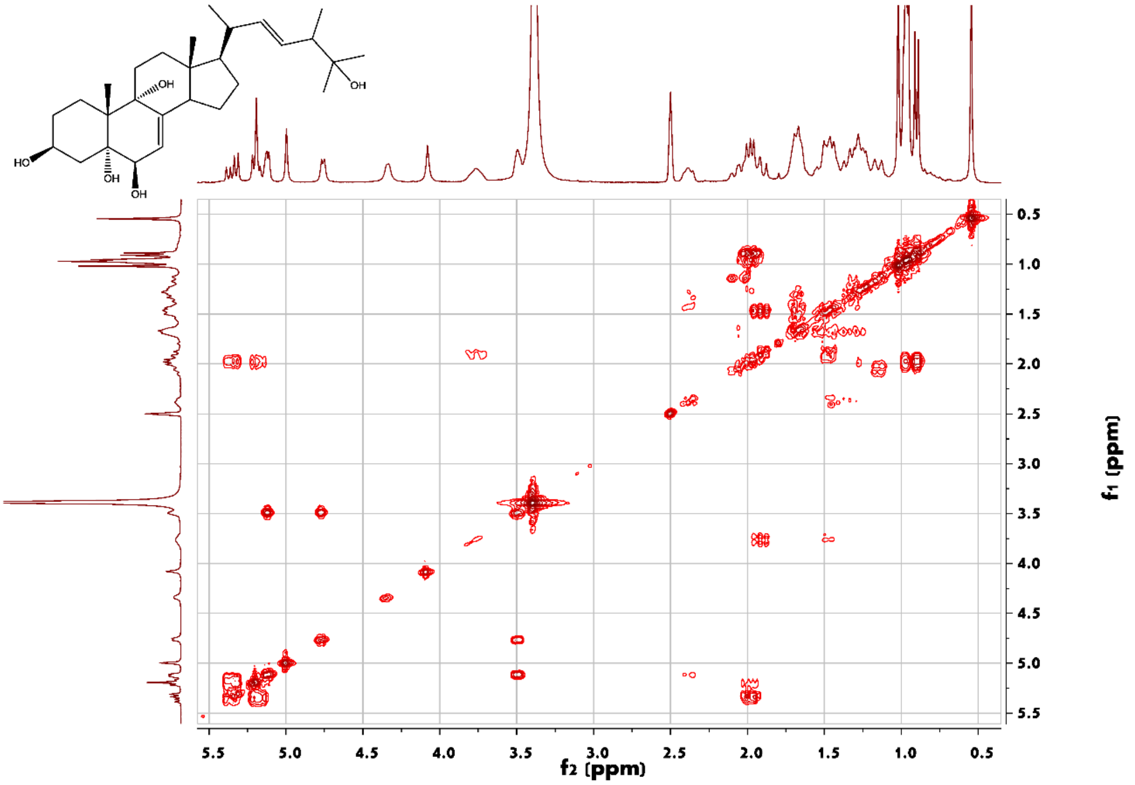

Figure S19. COSY spectrum of compound **2** (300 MHz in DMSO-*d*6).

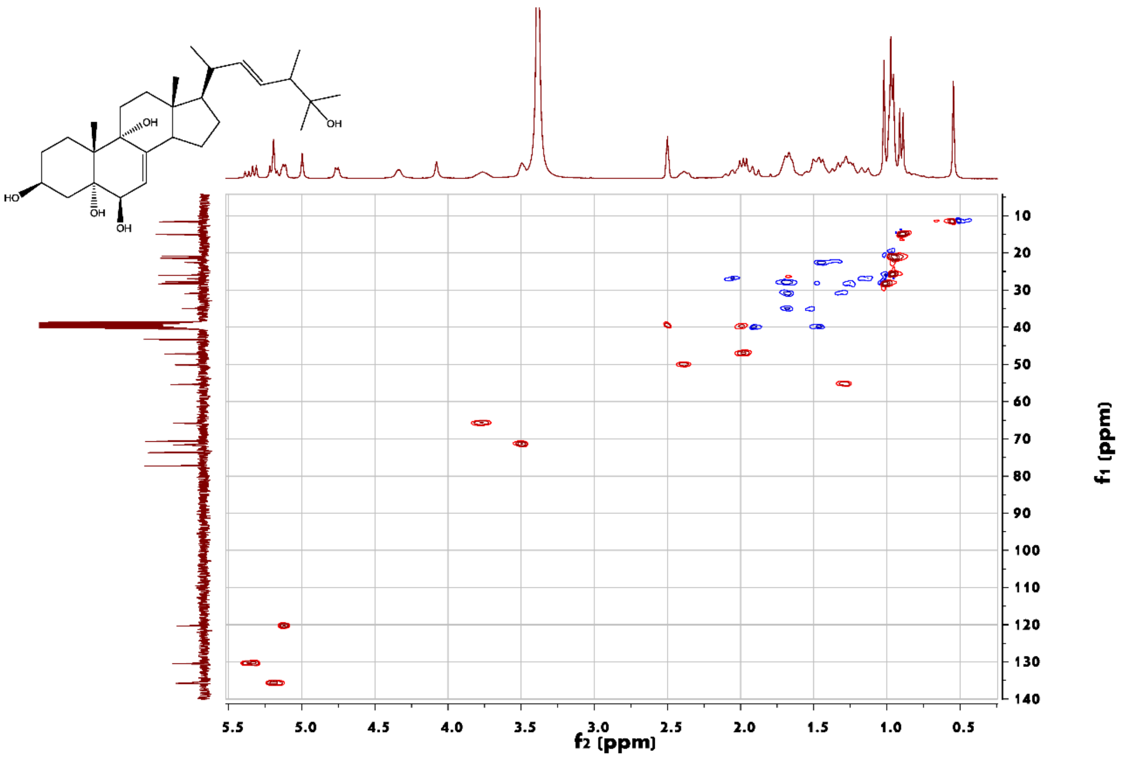

Figure S20. HMQC spectrum of compound **2** (300 MHz in DMSO-*d*6).

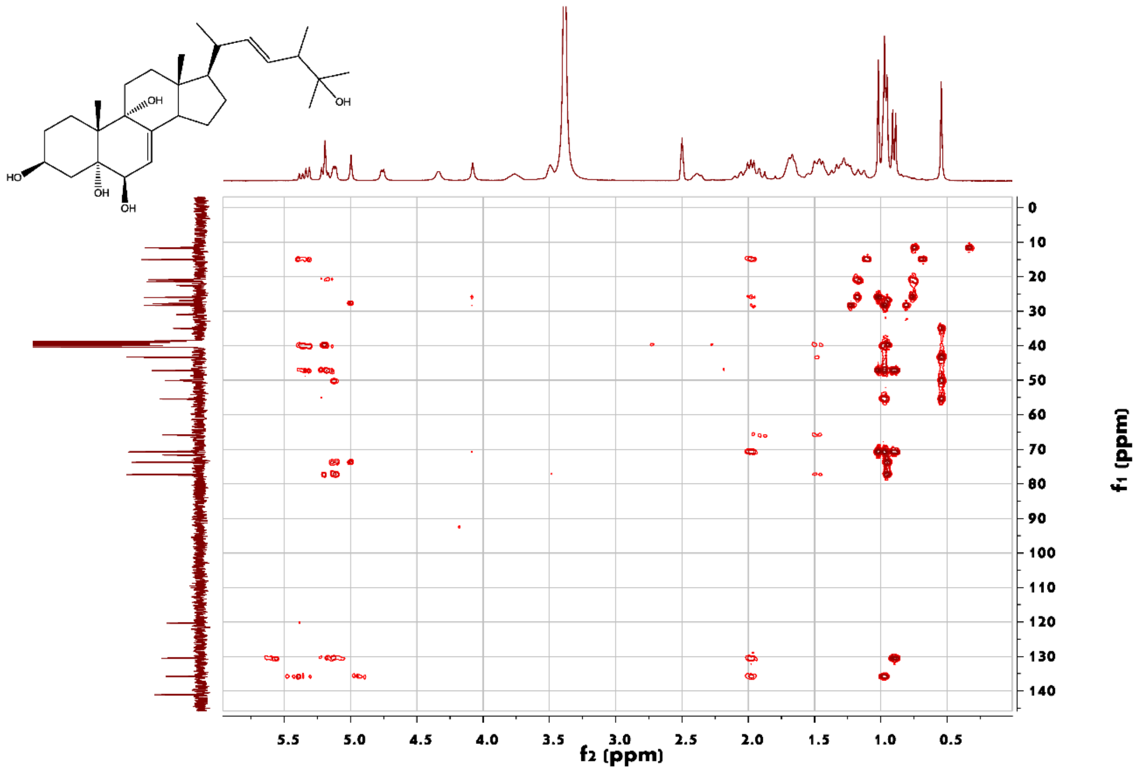

Figure S21. HMBC spectrum of compound **2** (300 MHz in DMSO-*d6*).

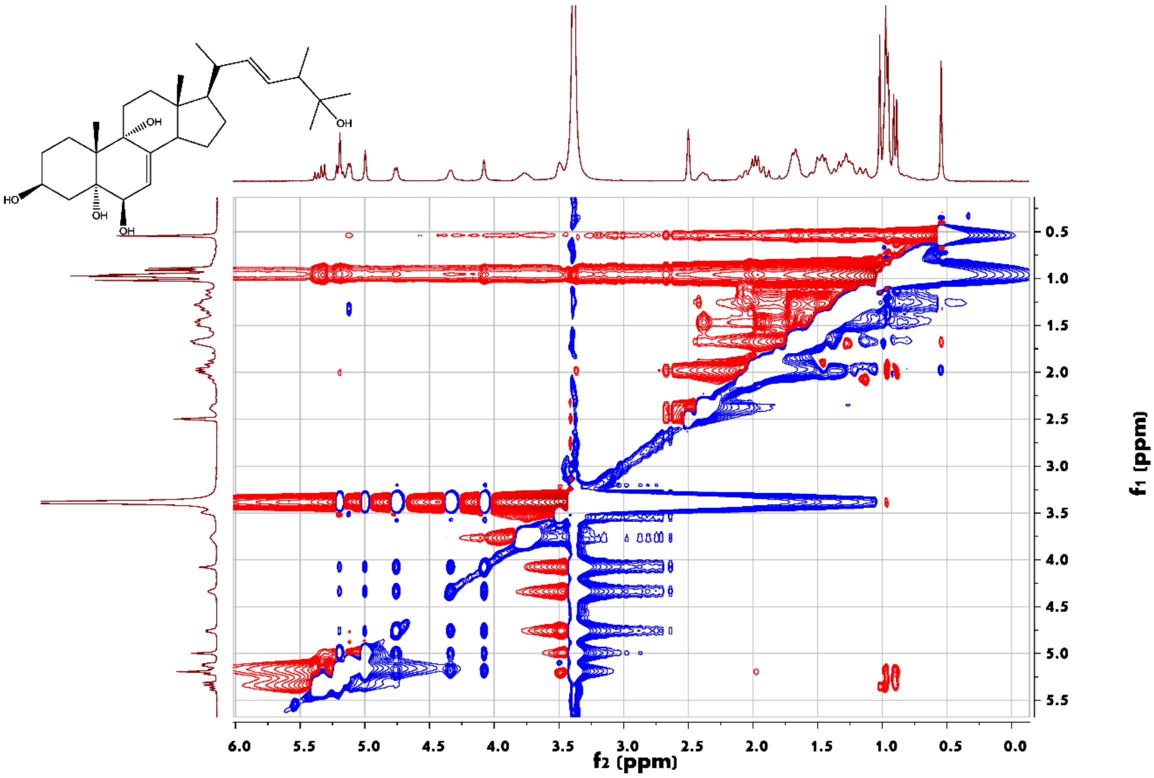

Figure S22. NOESY spectrum of compound **2** (300 MHz in DMSO-*d6*).

Compound 3

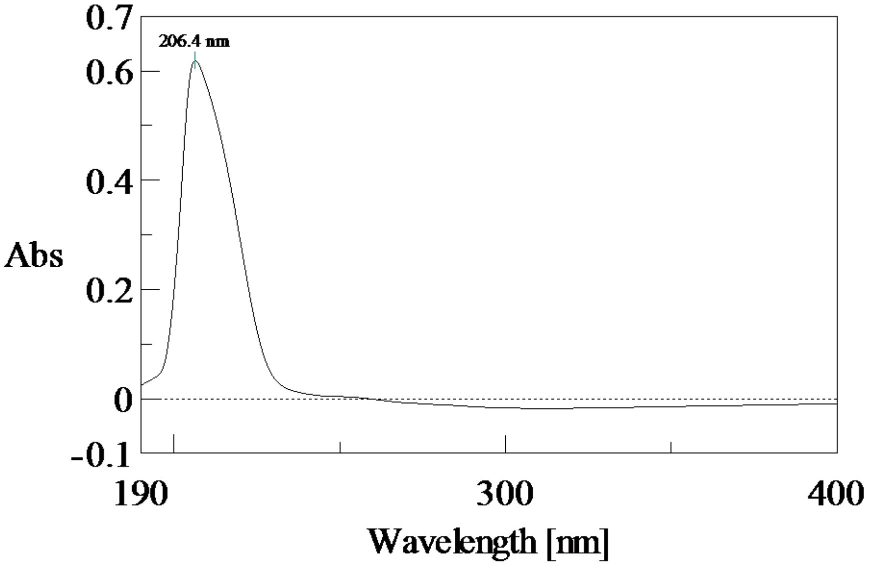

Figure S23. UV spectrum of compound **3** in MeOH.

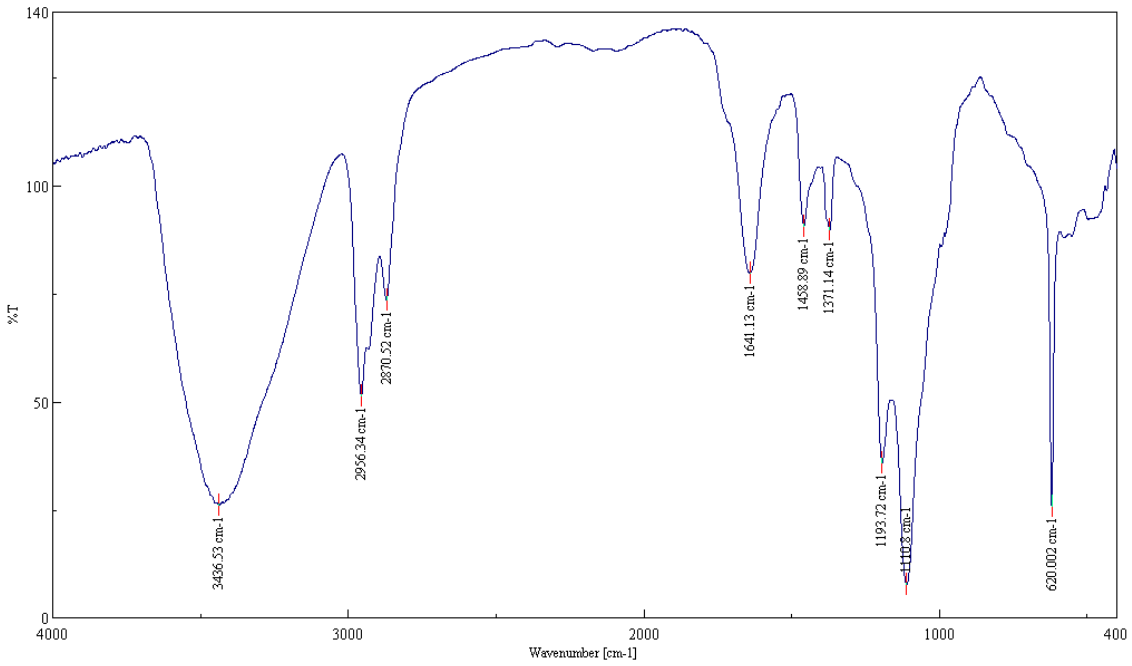

Figure S24. IR spectrum of compound **3**.

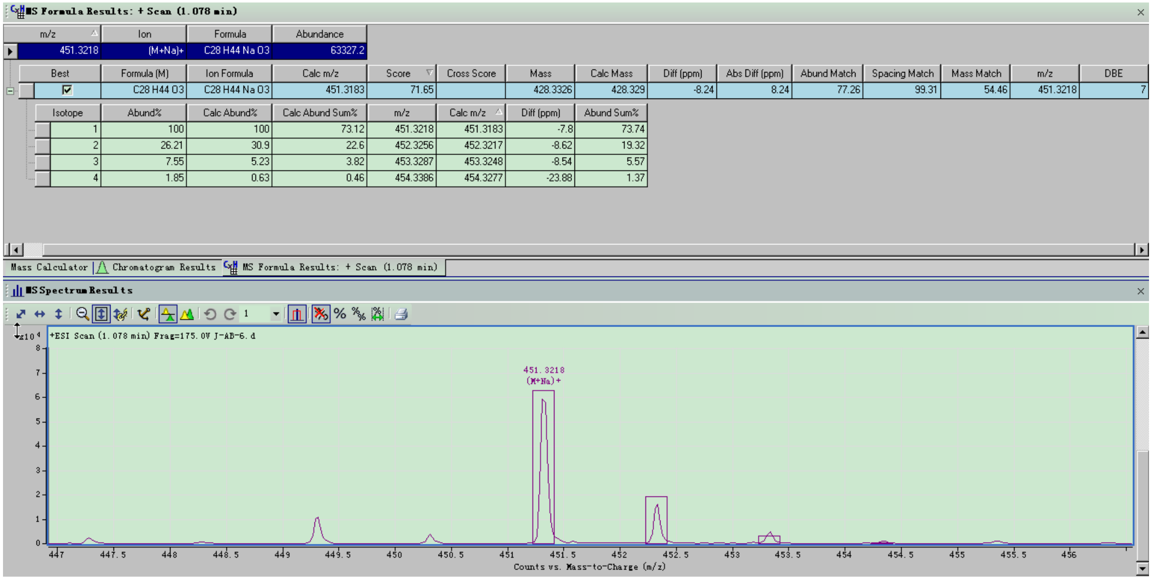

Figure S25. HR-MS of compound **3**.

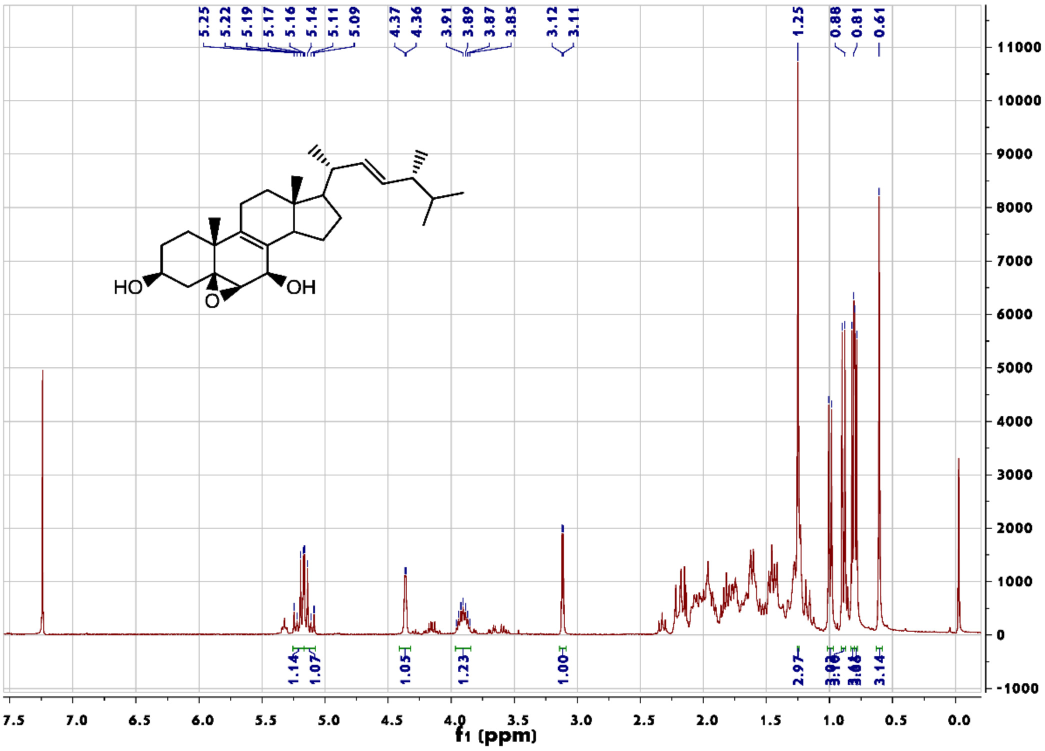

Figure S26. ^{1}H-NMR spectrum of compound **3** (300 MHz in $CDCl_3$).

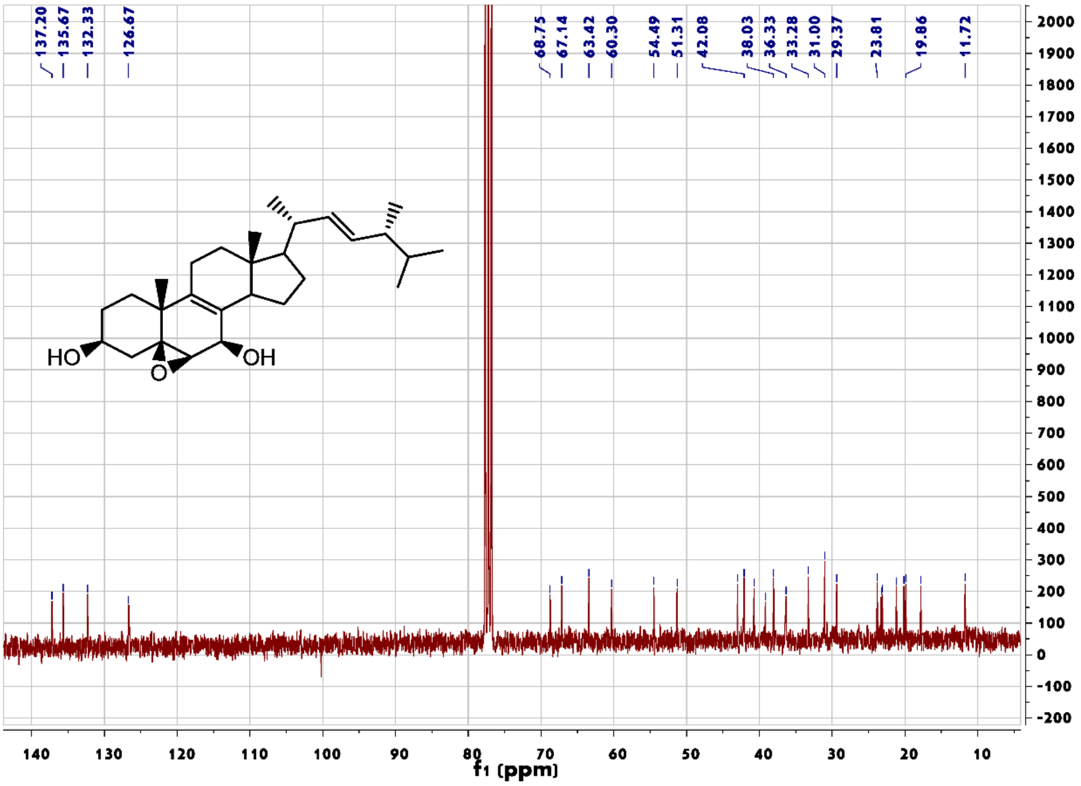

Figure S27. ^{13}C-NMR spectrum of compound **3** (75 MHz in CDCl3).

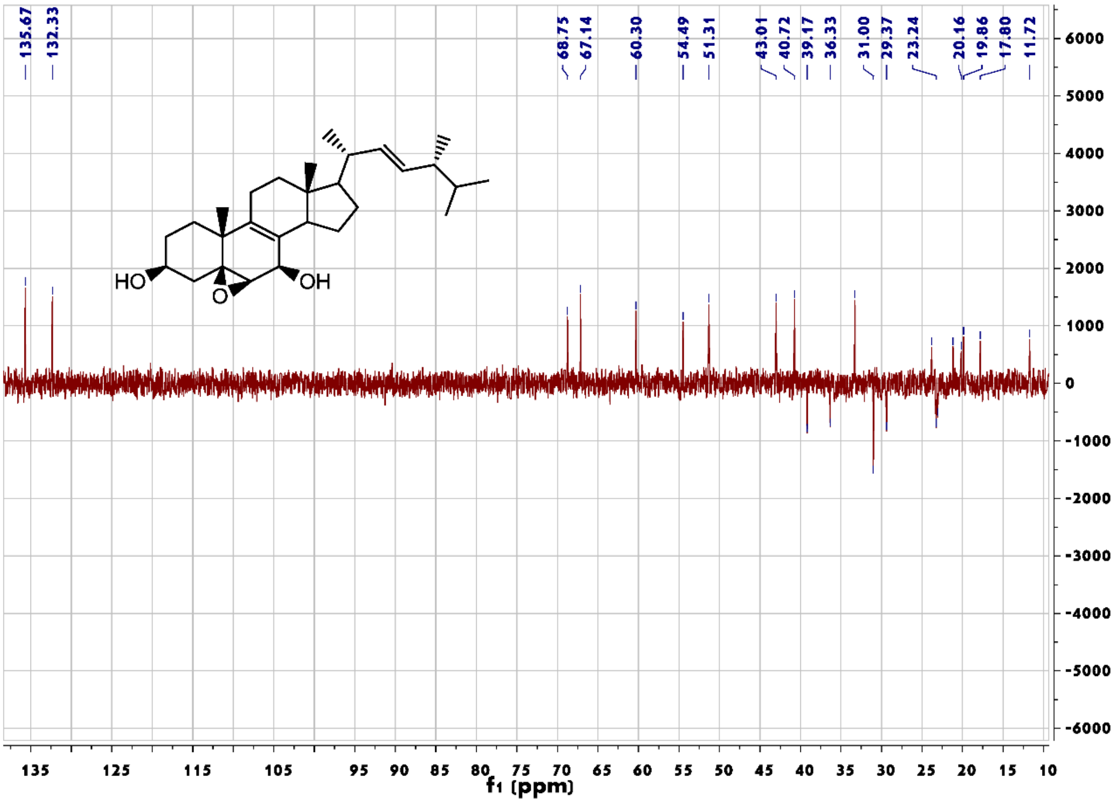

Figure S28. DEPT spectrum of compound **3** (75 MHz in CDCl3).

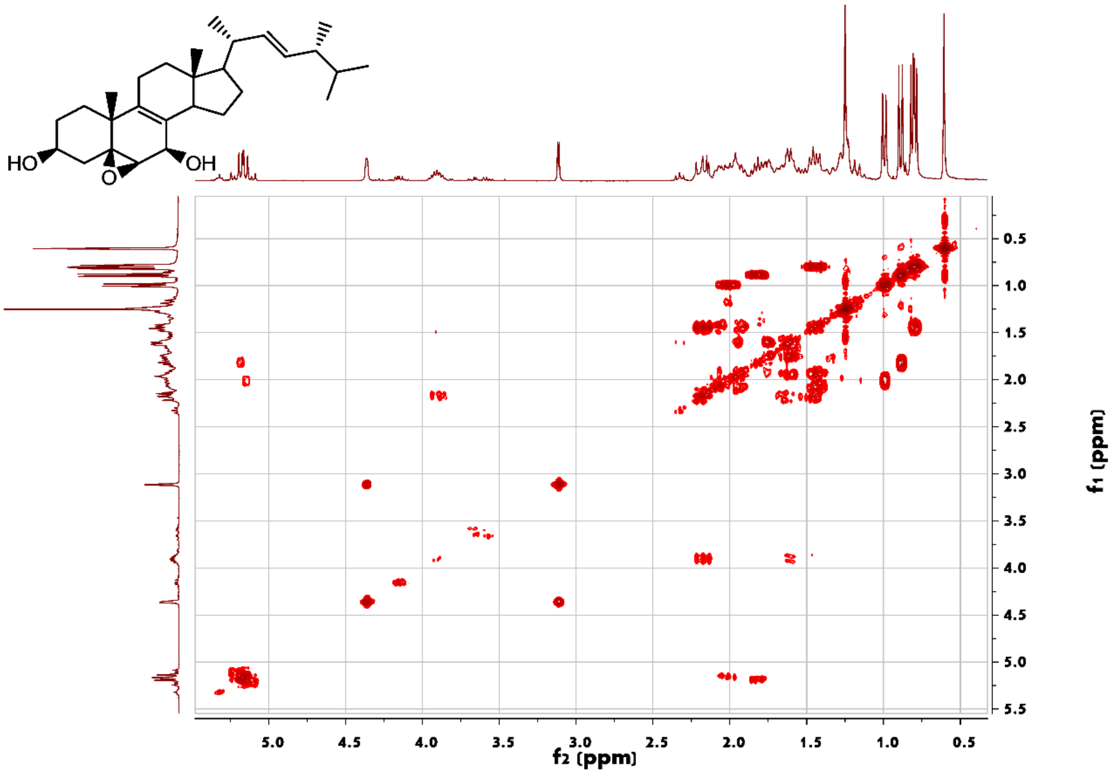

Figure S29. COSY spectrum of compound **3** (300 MHz in CDCl3).

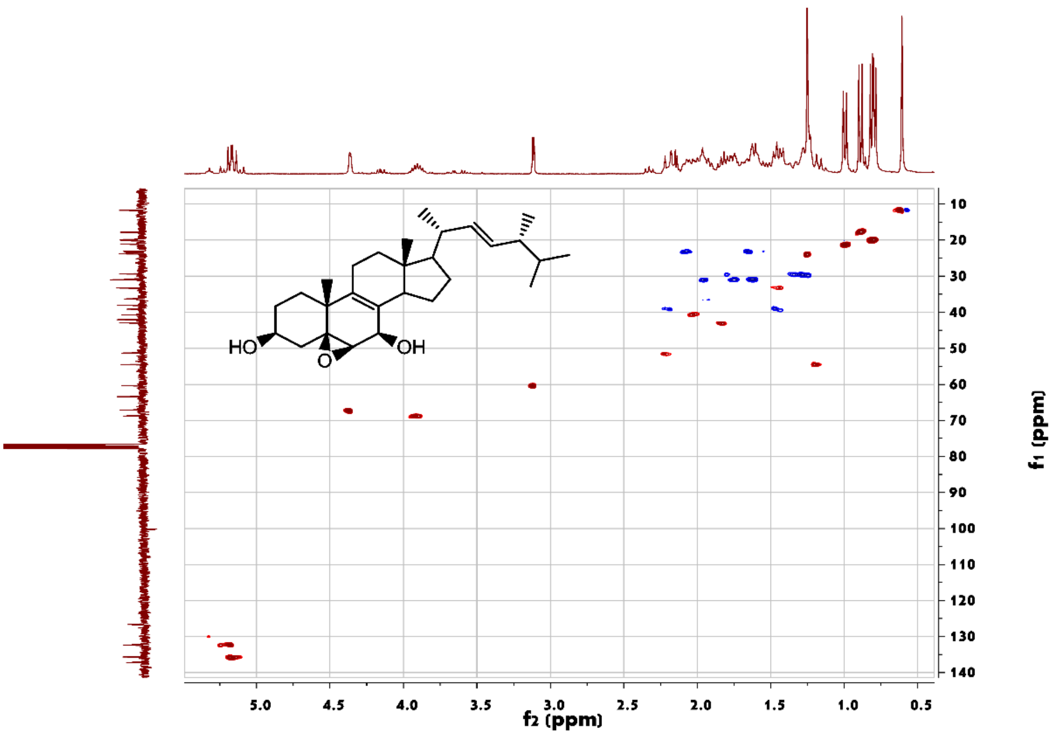

Figure S30. HMQC spectrum of compound **3** (300 MHz in CDCl3).

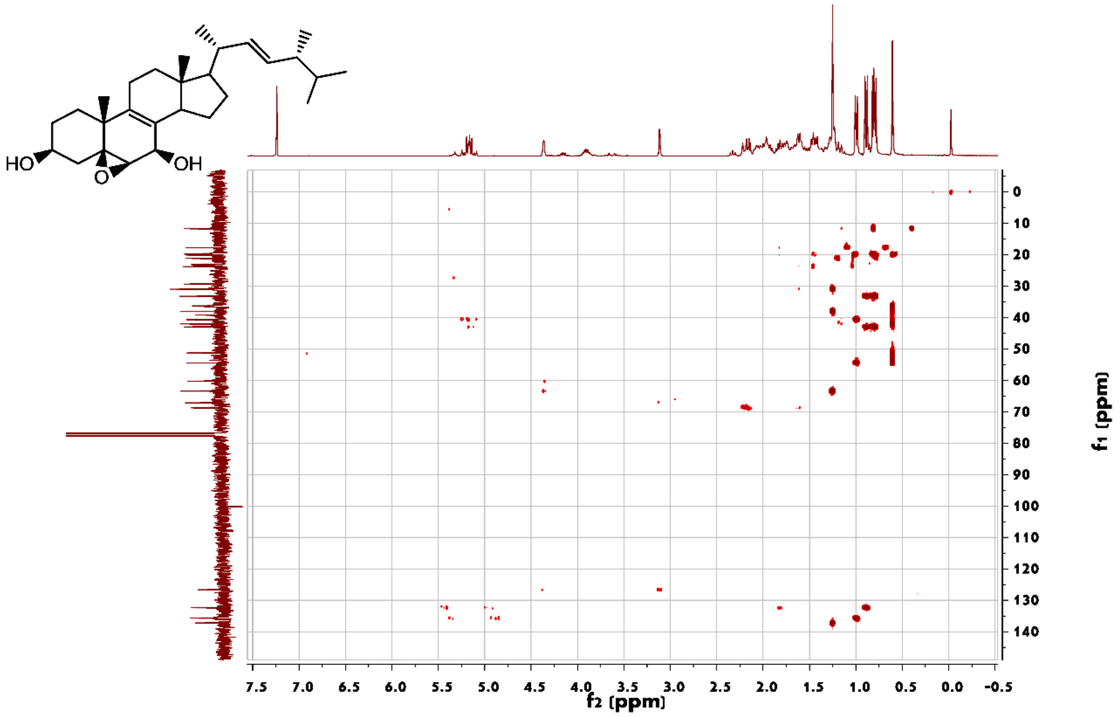

Figure S31. HMBC spectrum of compound **3** (300 MHz in CDCl3).

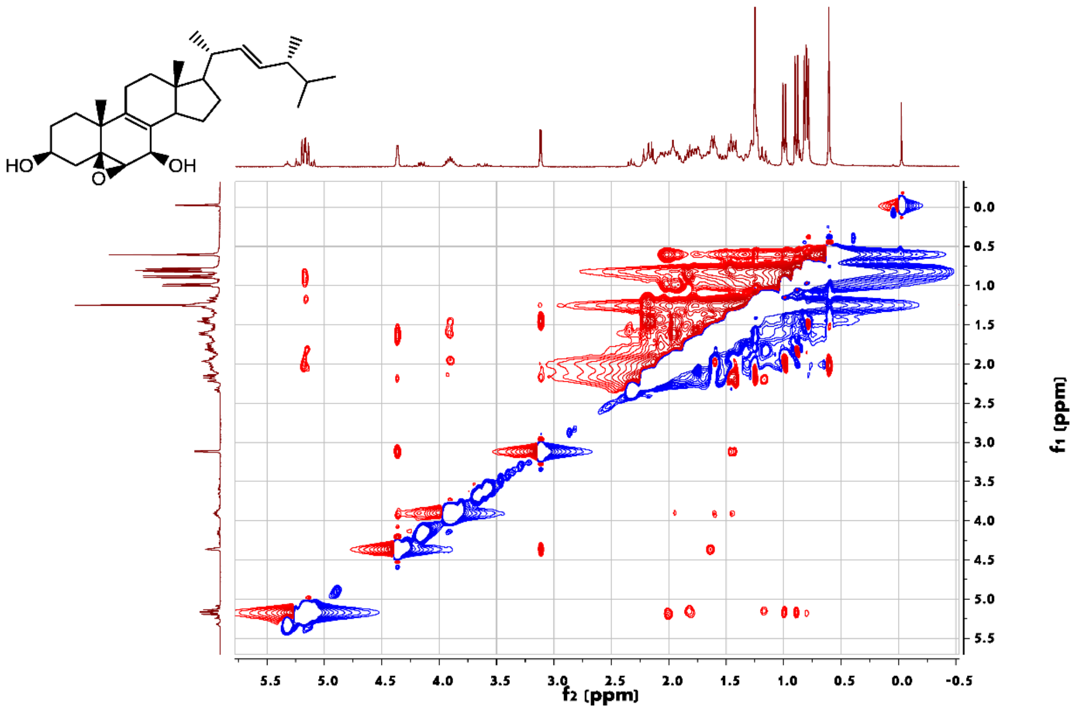

Figure S32. NOESY spectrum of compound **3** (300 MHz in CDCl3).

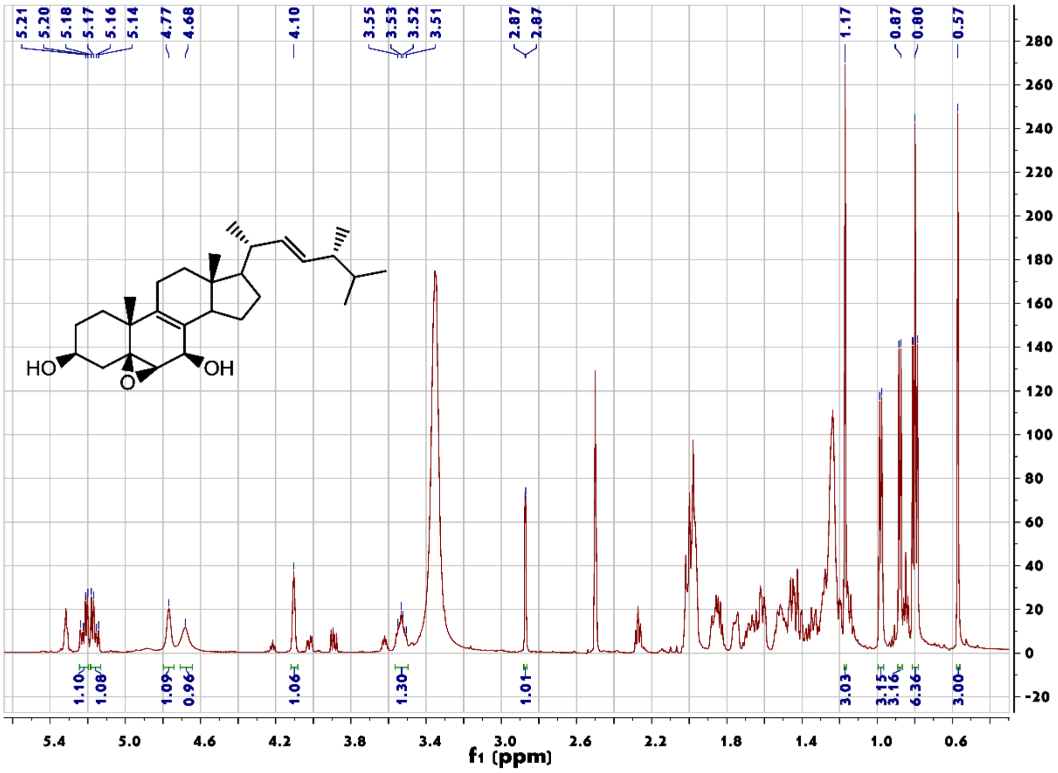

Figure S33. ^{1}H-NMR spectrum of compound **3** (600 MHz in DMSO-*d*6).

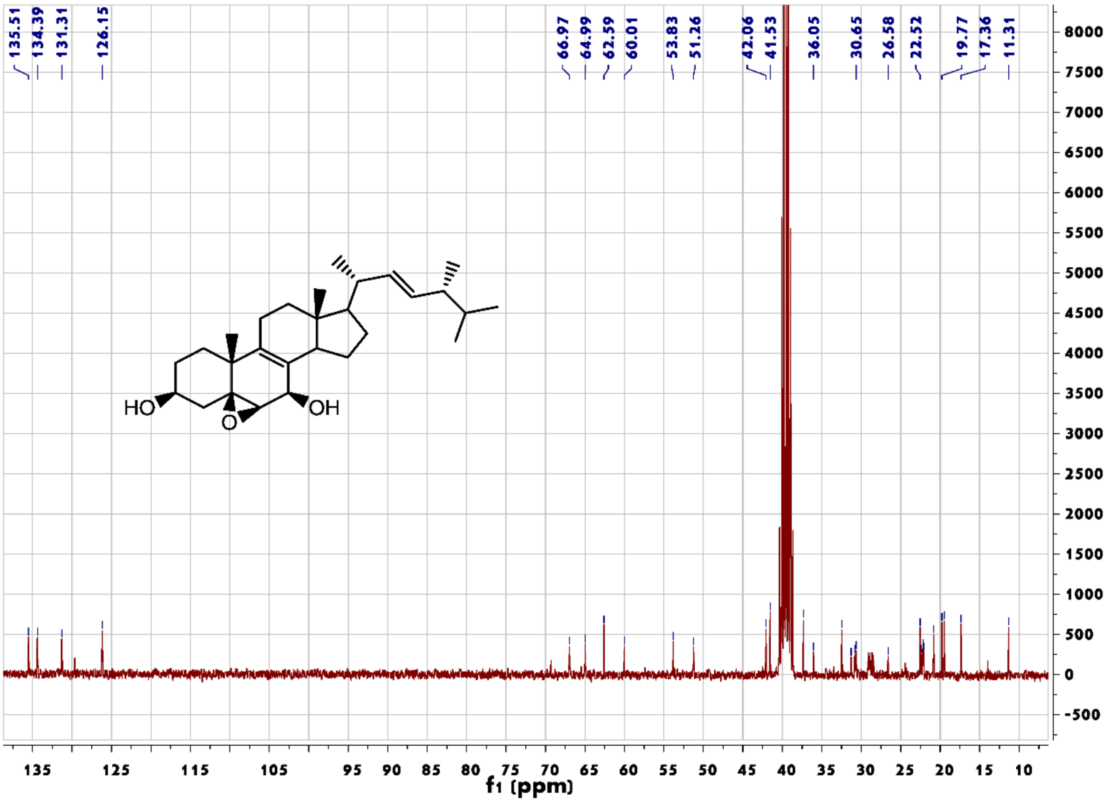

Figure S34. ^{13}C-NMR spectrum of compound **3** (150 MHz in DMSO-*d*6).

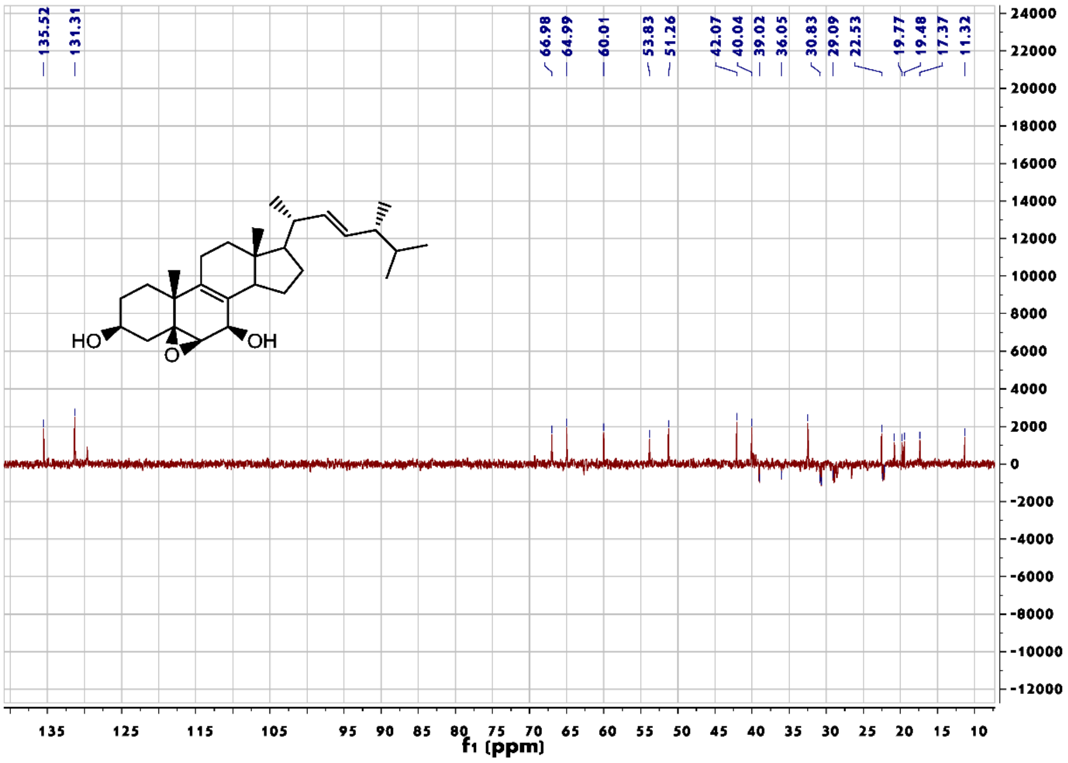

Figure S35. DEPT spectrum of compound **3** (150 MHz in DMSO-*d6*).

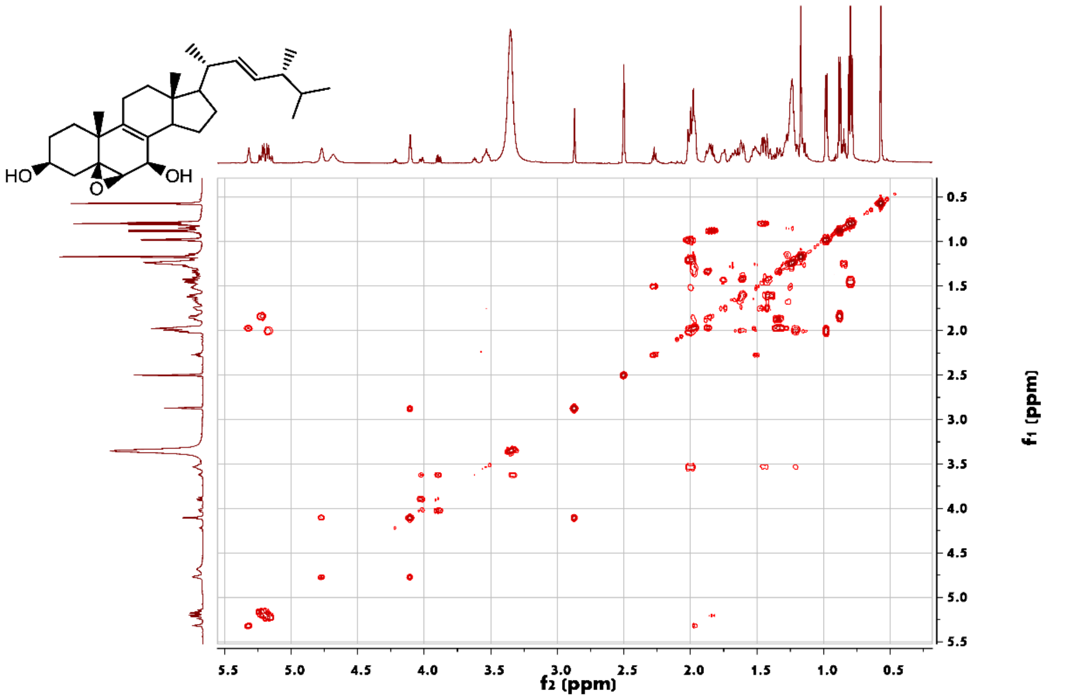

Figure S36. COSY spectrum of compound **3** (600 MHz in DMSO-*d6*).

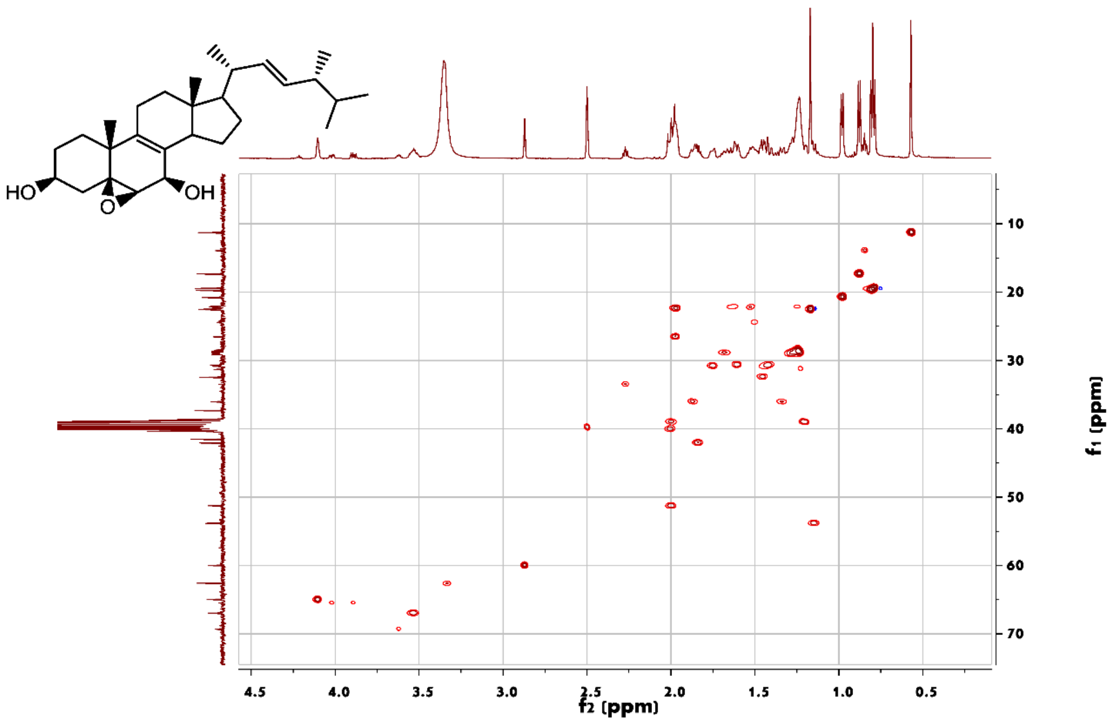

Figure S37. HMQC spectrum of compound **3** (600 MHz in DMSO-*d*6).

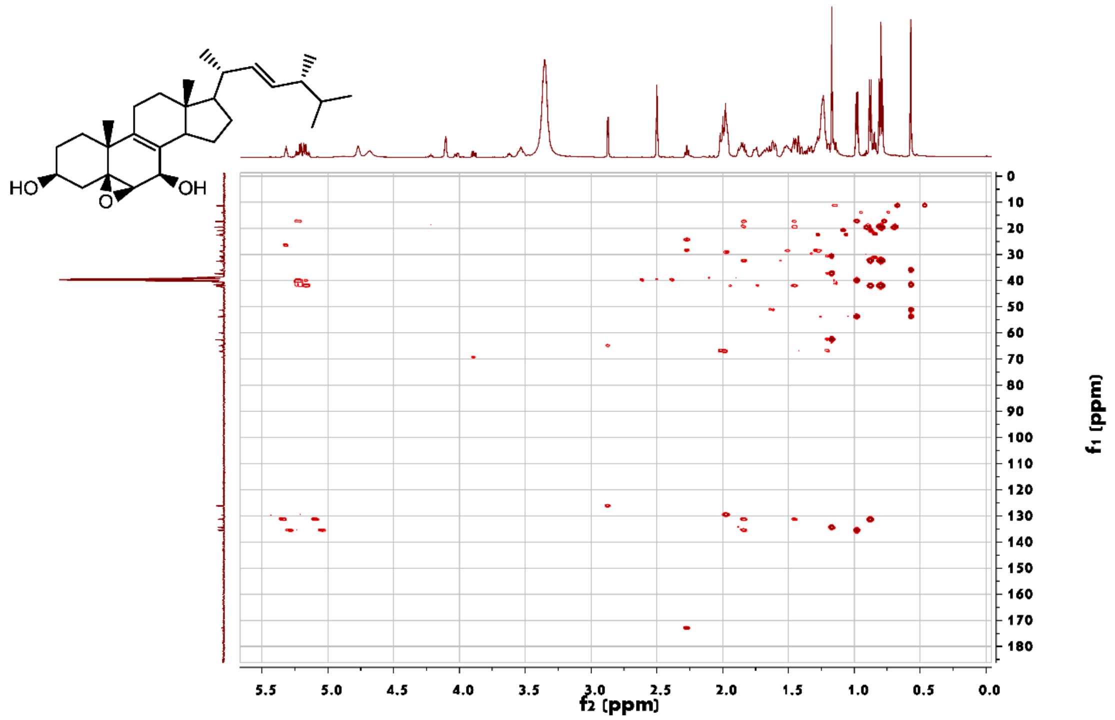

Figure S38. HMBC spectrum of compound **3** (600 MHz in DMSO-*d*6).

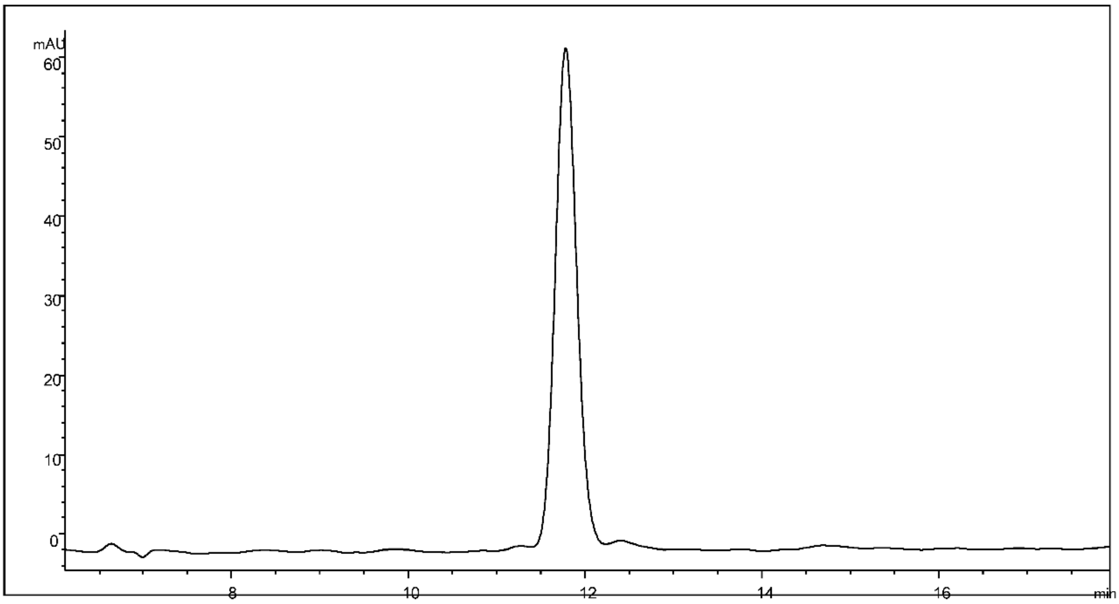

Figure S39. HPLC profile of compound **1** (80% MeOH at 210 nm).

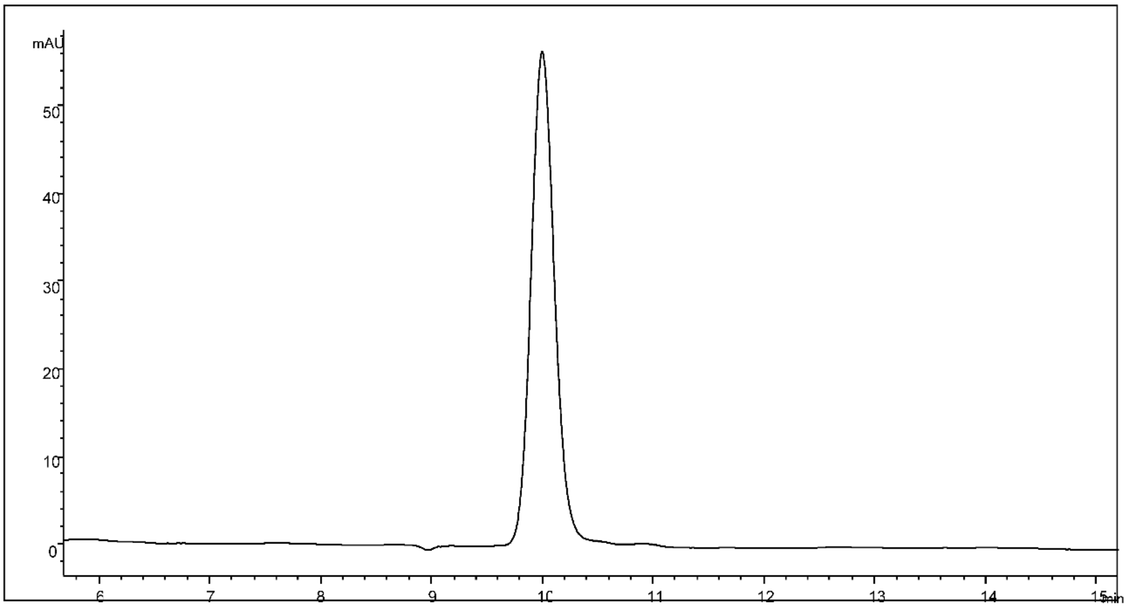

Figure S40. HPLC profile of compound **2** (75% MeOH at 210 nm).

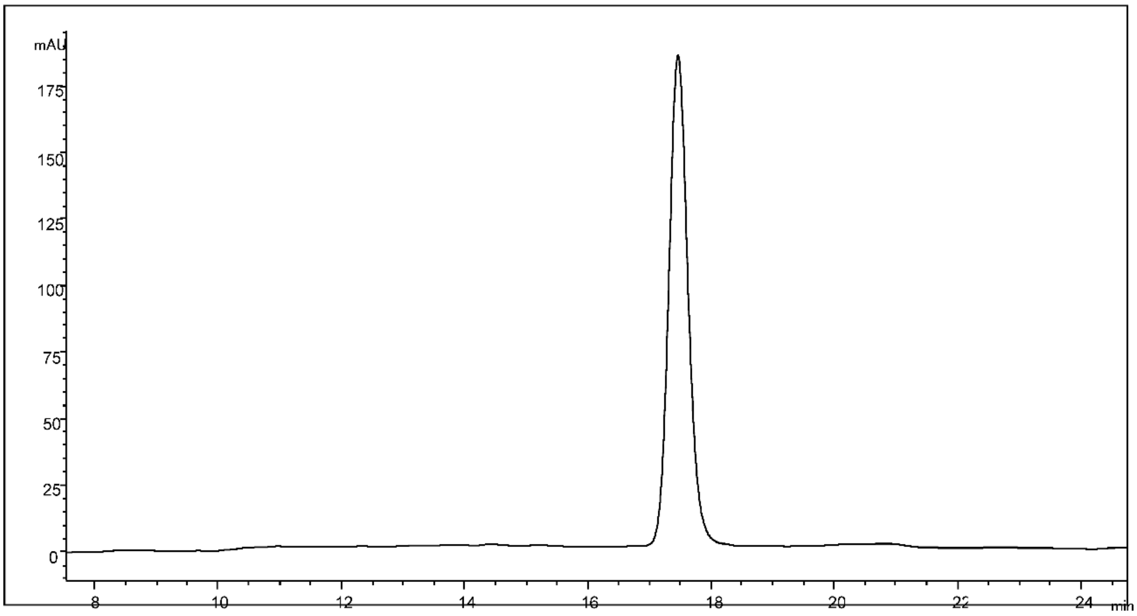

Figure S41. HPLC profile of compound **3** (90% MeOH at 210 nm).

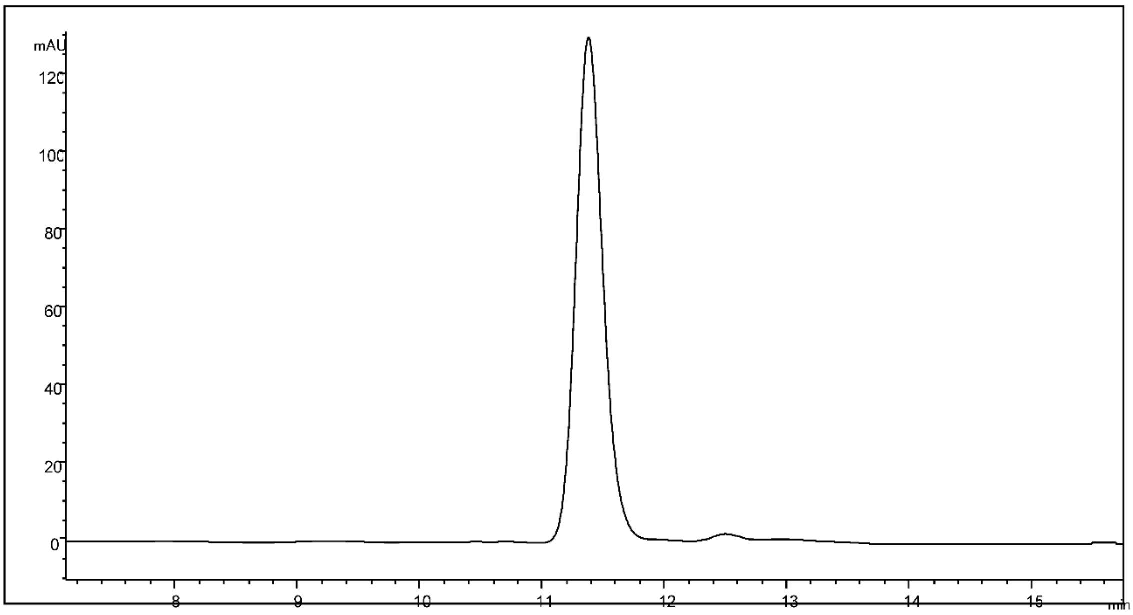

Figure S42. HPLC profile of compound **4** (80% MeOH at 210 nm).

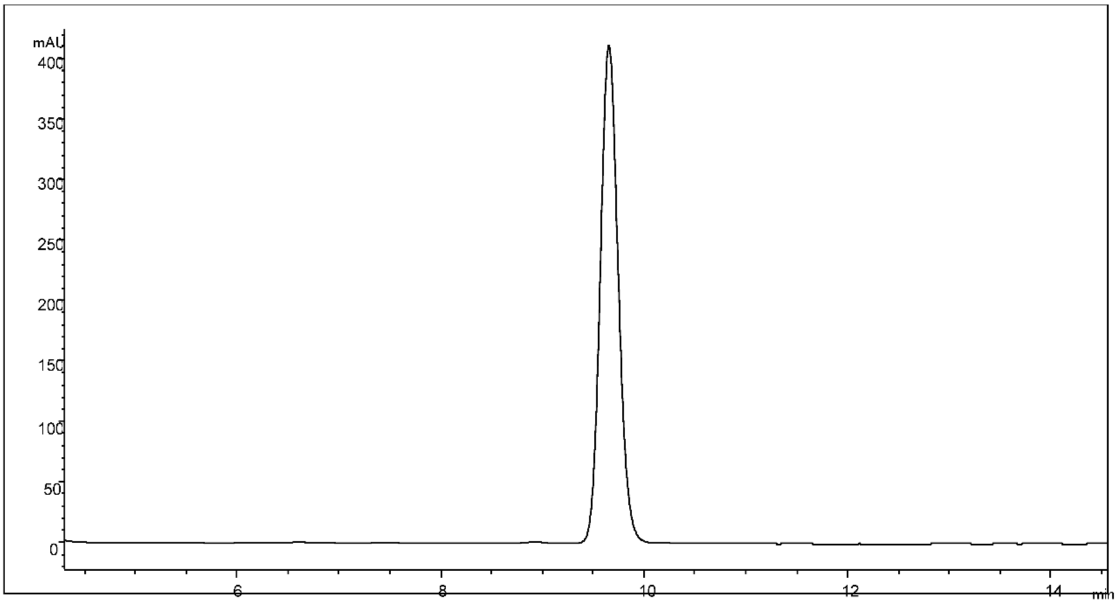

Figure S43. HPLC profile of compound **5** (95% MeOH at 210 nm).

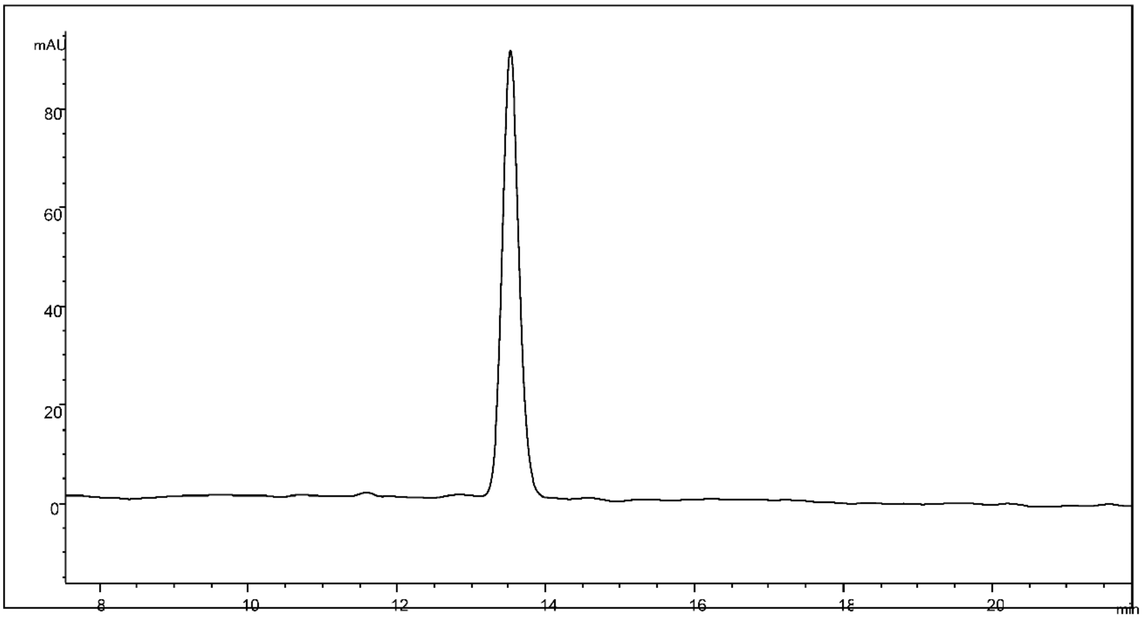

Figure S44. HPLC profile of compound **6** (95% MeOH at 210 nm).

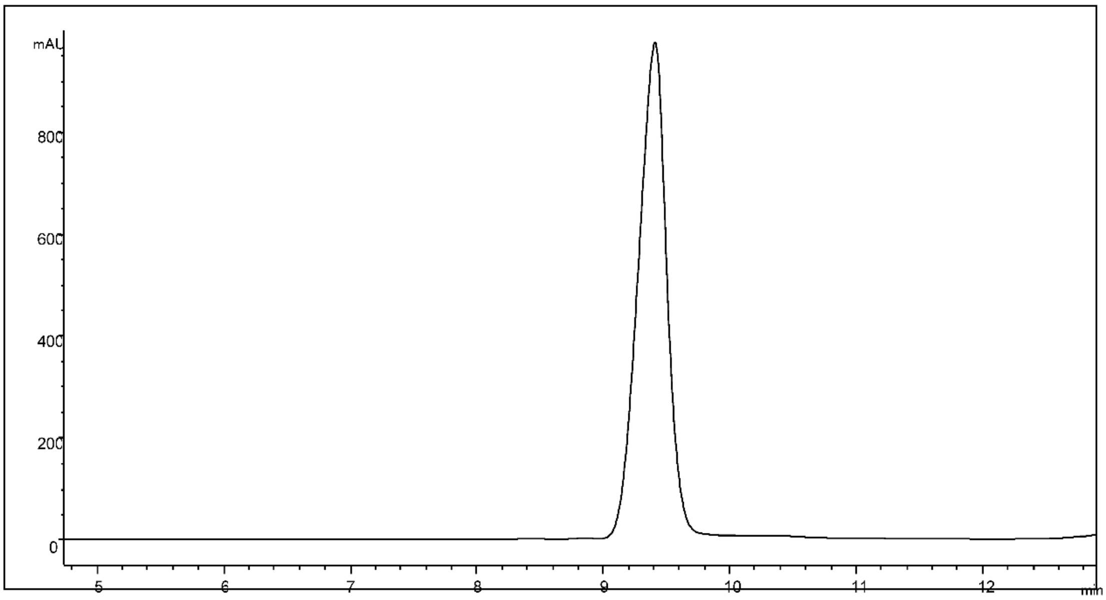

Figure S45. HPLC profile of compound **7** (90% MeOH at 210 nm).

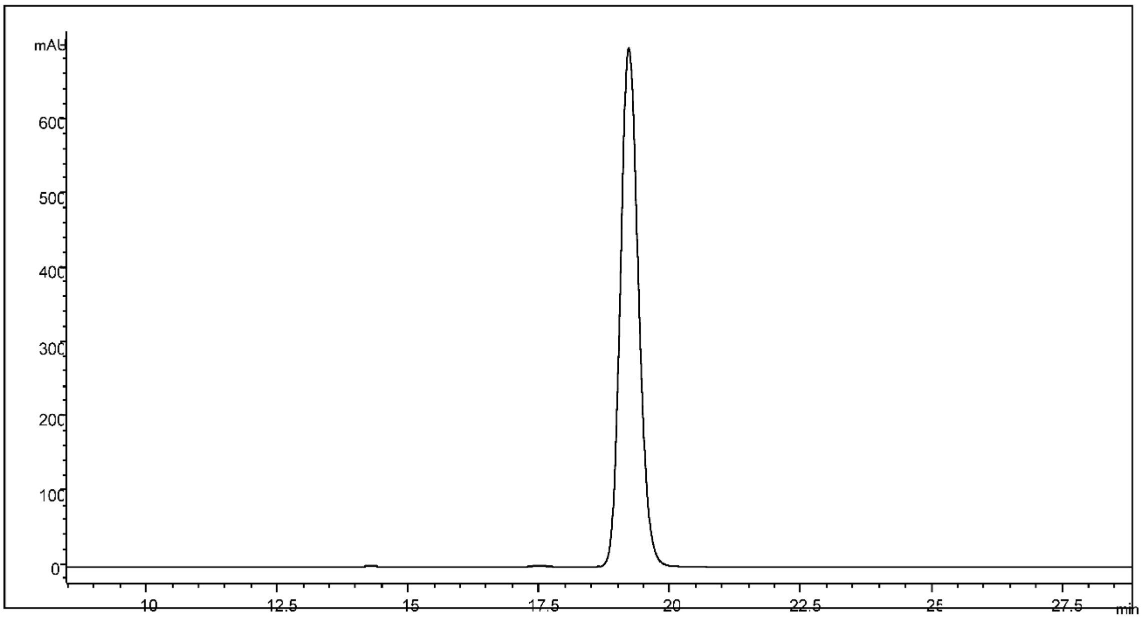

Figure S46. HPLC profile of compound **8** (80% MeOH at 210 nm).

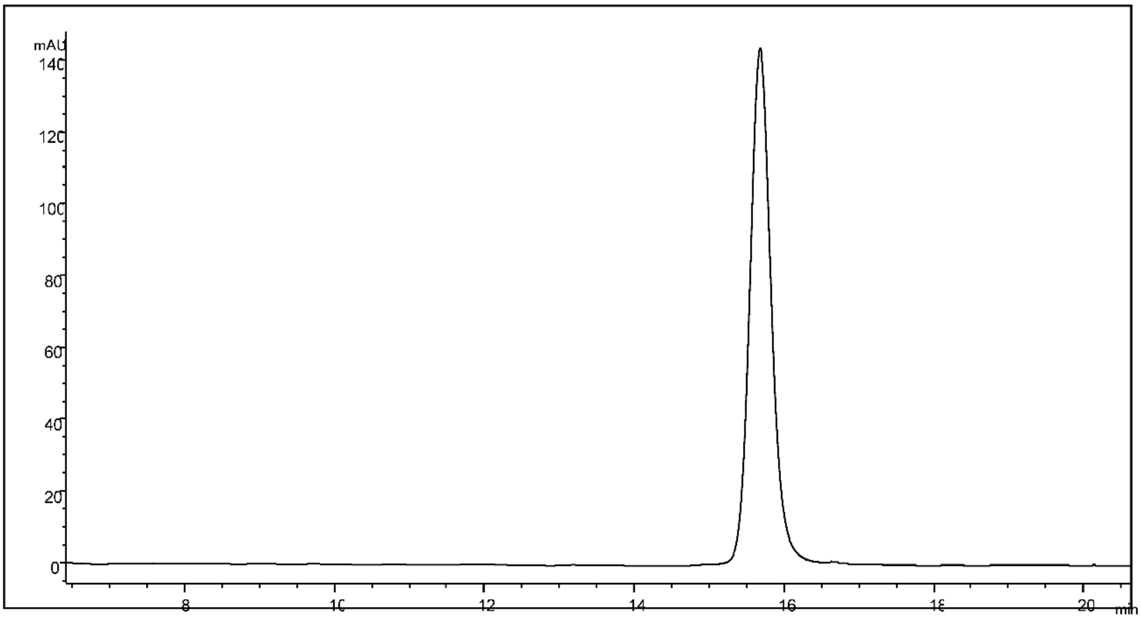

Figure S47. HPLC profile of compound **11** (90% MeOH at 210 nm).

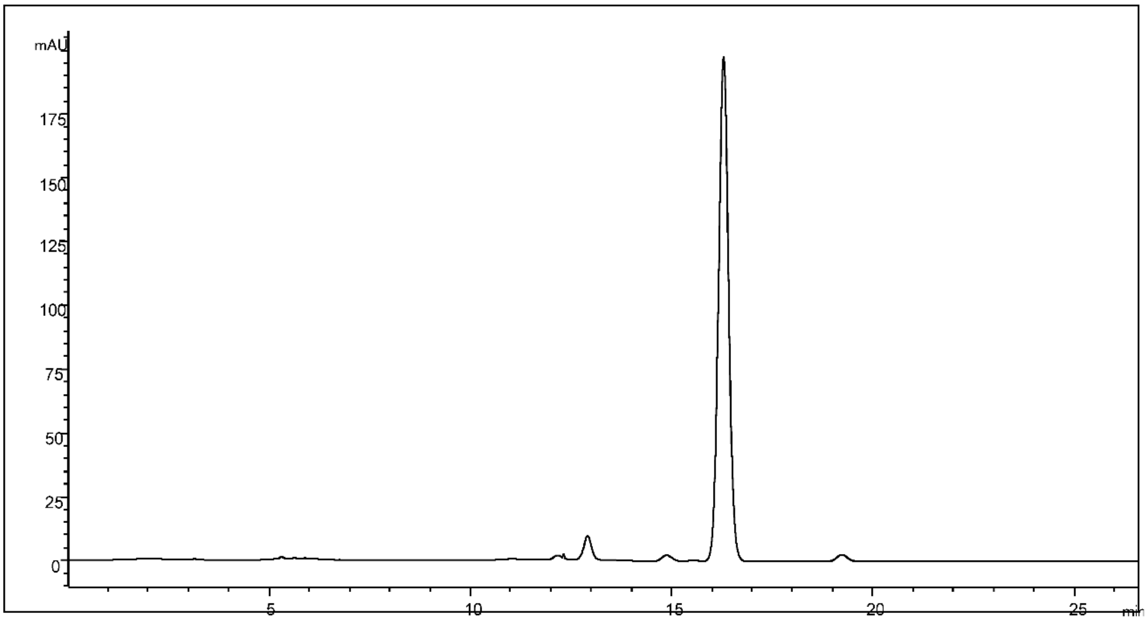

Figure S48. HPLC profile of compound **12** (100% MeOH at 280 nm).

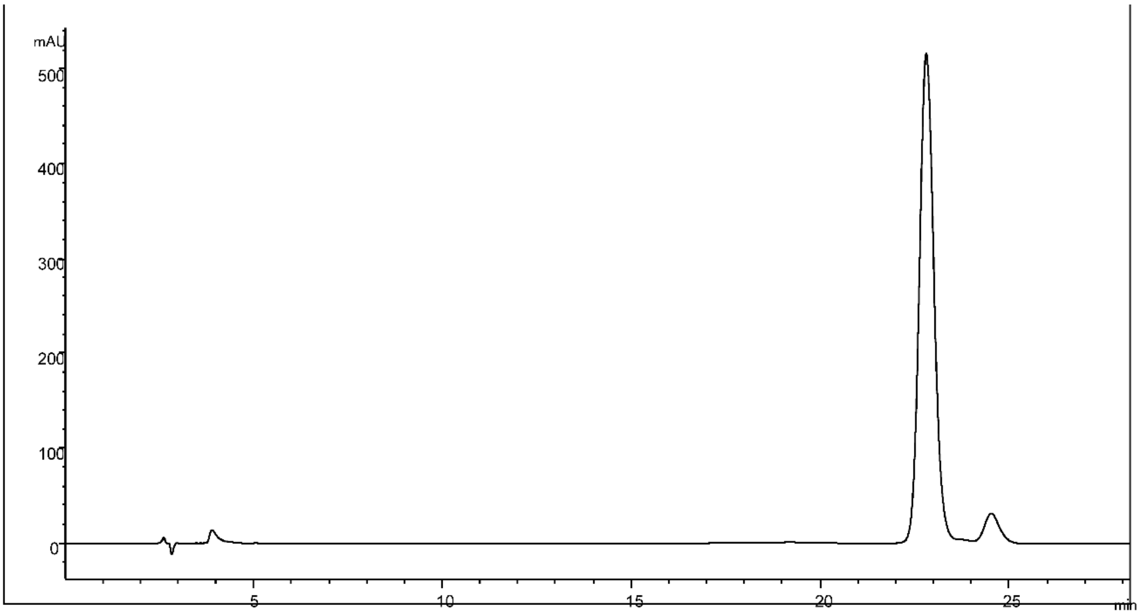

Figure S49. HPLC profile of compound **13** (90% MeOH at 210nm).

Table S1. Characteristics of compounds **1**–**8**, **11** and **12**.

Compounds	Retention Time (min)	Purity	Wavelength
**1**	11.773	97.60%	210
**2**	10.004	98.97%	210
**3**	19.003	99.00%	210
**4**	11.376	96.805%	210
**5**	9.657	99.09%	210
**6**	13.524	99.12%	210
**7**	9.406	93.57%	210
**8**	19.227	98.87%	210
**11**	15.686	98.07%	210
**12**	15.803	97.893	280
**13**	22.797	93.774	210

Table S2. ^{13}C-NMR data (δ) of **4**–**13** (75 MHz).

No.	4 [a]	5 [a]	6 [a,*]	7 [a]	8 [b]	9 [b]	10 [a,*]	11 [a,*]	12 [b]	13 [b]
**1**	31.2	31.2	31.4	26.9	30.4	31.1	30.7	31.7	38.6	31.4
**2**	32.5	32.5	30.9	31.0	31.0	32.2	31.0	31	32.0	33.2
**3**	65.9	66.0	65.6	65.8	68.8	68.7	65.1	65.1	70.7	68.9
**4**	40.2	40.0	39.2	40.2	39.3	39.6	40.5	40.9	41.0	39.5
**5**	74.5	74.5	74.5	73.7	65.9	67.8	74.5	76.3	140.0	65.1
**6**	72.1	72.2	69.1	71.6	62.8	61.3	77.9	76.0	116.5	60.5
**7**	119.5	119.5	120.6	120.3	67.0	65.1	71.6	71.6	119.8	63.9
**8**	139.7	139.7	139.7	141.1	127.1	125.2	127.2	127.0	141.6	126.9
**9**	42.3	42.3	42.6	77.3	134.7	38.7	136.2	36.4	46.3	39.8
**10**	36.7	36.6	38.1	40.0	38.2	35.8	40.2	39.8	37.2	34.9
**11**	27.7	21.3	20.6	26.9	23.6	19.0	23.0	18.8	21.3	19.5
**12**	39.0	38.9	38.9	35.0	35.9	36.6	35.9	36.6	39.3	37.3
**13**	43.0	43.0	43.1	43.3	42.3	42.9	41.3	42.4	43.0	43.8
**14**	54.2	54.2	54.2	50.1	49.8	152.5	49.4	147.6	54.6	151.9
**15**	22.6	22.6	22.4	22.6	24.1	24.9	28.6	24.7	23.2	26.1
**16**	27.7	27.8	27.9	27.8	29.2	27.2	22.7	27.2	28.5	28.2
**17**	55.3	55.3	55.2	55.4	53.8	56.8	53.9	56.0	55.8	55.6
**18**	12.1	12.0	12.0	11.7	11.5	17.6	11.2	18.0	12.3	17.9
**19**	17.7	17.7	17.4	21.4	23.0	16.5	22.9	17.5	16.5	17.1
**20**	40.1	40.0	39.8	39.8	40.6	39.3	40.0	38.5	42.9	40.6
**21**	20.9	21	21	21.0	21.2	21.2	20.9	21.2	21.3	21.3
**22**	135.7	135.4	135.4	135.5	135.8	135.3	135.5	135.5	135.8	135.4
**23**	130.5	131.4	131.4	131.4	132.2	132.2	131.3	131.5	132.2	132.5
**24**	47.2	42.0	41.9	42.1	43.0	42.8	42.1	42.1	40.4	43.1
**25**	70.7	32.5	32.5	32.5	33.3	33.1	32.5	32.5	33.3	33.3
**26**	26.0	19.5	19.5	19.5	19.9	19.7	19.5	19.5	19.9	19.9
**27**	28.3	19.8	19.8	19.8	20.2	20.0	19.8	19.8	20.2	20.2
**28**	15.0	17.3	17.3	17.4	17.9	18.1	17.4	17.4	17.8	17.9

[a] Measured in DMSO-*d*6; [b] Measured in CDCl3; * Measured in solvent different from literature.
